# Antibiotic Selection Pressure and Macrolide Resistance in Nasopharyngeal *Streptococcus pneumoniae:* A Cluster-Randomized Clinical Trial

**DOI:** 10.1371/journal.pmed.1000377

**Published:** 2010-12-14

**Authors:** Alison H. Skalet, Vicky Cevallos, Berhan Ayele, Teshome Gebre, Zhaoxia Zhou, James H. Jorgensen, Mulat Zerihun, Dereje Habte, Yared Assefa, Paul M. Emerson, Bruce D. Gaynor, Travis C. Porco, Thomas M. Lietman, Jeremy D. Keenan

**Affiliations:** 1 Francis I. Proctor Foundation for Research in Opthalmology, University of California, San Francisco, San Francisco, California, United States of America; 2 Department of Ophthalmology, University of California, San Francisco, San Francisco, California, United States of America; 3 The Carter Center, Addis Ababa, Ethiopia; 4 University of Texas Health Sciences Center, San Antonio, Texas, United States of America; 5 Department of Ophthalmology, University of Gondar, Ethiopia; 6 The Carter Center, Atlanta, Georgia, United States of America; 7 Department of Epidemiology & Biostatistics, University of California, San Francisco, San Francisco, California, United States of America; 8 Institute for Global Health, University of California, San Francisco, San Francisco, California, United States of America; Brown University School of Medicine, United States of America

## Abstract

**Background:**

It is widely thought that widespread antibiotic use selects for community antibiotic resistance, though this has been difficult to prove in the setting of a community-randomized clinical trial. In this study, we used a randomized clinical trial design to assess whether macrolide resistance was higher in communities treated with mass azithromycin for trachoma, compared to untreated control communities.

**Methods and Findings:**

In a cluster-randomized trial for trachoma control in Ethiopia, 12 communities were randomized to receive mass azithromycin treatment of children aged 1–10 years at months 0, 3, 6, and 9. Twelve control communities were randomized to receive no antibiotic treatments until the conclusion of the study. Nasopharyngeal swabs were collected from randomly selected children in the treated group at baseline and month 12, and in the control group at month 12. Antibiotic susceptibility testing was performed on *Streptococcus pneumoniae* isolated from the swabs using Etest strips. In the treated group, the mean prevalence of azithromycin resistance among all monitored children increased from 3.6% (95% confidence interval [CI] 0.8%–8.9%) at baseline, to 46.9% (37.5%–57.5%) at month 12 (*p* = 0.003). In control communities, azithromycin resistance was 9.2% (95% CI 6.7%–13.3%) at month 12, significantly lower than the treated group (*p*<0.0001). Penicillin resistance was identified in 0.8% (95% CI 0%–4.2%) of isolates in the control group at 1 year, and in no isolates in the children-treated group at baseline or 1 year.

**Conclusions:**

This cluster-randomized clinical trial demonstrated that compared to untreated control communities, nasopharyngeal pneumococcal resistance to macrolides was significantly higher in communities randomized to intensive azithromycin treatment. Mass azithromycin distributions were given more frequently than currently recommended by the World Health Organization's trachoma program. Azithromycin use in this setting did not select for resistance to penicillins, which remain the drug of choice for pneumococcal infections.

**Trial registration:**

www.ClinicalTrials.gov NCT00322972

*Please see later in the article for the Editors' Summary*

## Abstract 2

Jeremy Keenan and colleagues report that during a cluster-randomized clinical trial in Ethiopia, nasopharyngeal pneumococcal resistance to macrolides was significantly higher in communities randomized to receive azithromycin compared with untreated control communities.

## Editors' Summary

**Background:**

In 1928, Alexander Fleming discovered penicillin, the first antibiotic (a drug that kills bacteria). By the early 1940s, scientists were able to make large quantities of penicillin and, in the following decades, several other classes of powerful antibiotics were discovered. For example, erythromycin—the first macrolide antibiotic—was developed in the early 1950s. For a time, it looked like bacteria and the diseases that they cause had been defeated. But bacteria rapidly become resistant to antibiotics. Under the “selective pressure” of an antibiotic, bacteria that have acquired a random change in their DNA that allows them to survive in the antibiotic's presence outgrow nonresistant bacteria. What's more, bacteria can transfer antibiotic resistance genes between themselves. Nowadays, antibiotic resistance is a major public health concern. Almost every type of disease-causing bacteria has developed resistance to one or more antibiotic in clinical use and multi-drug resistant bacteria are causing outbreaks of potentially fatal diseases in hospitals and in the community.

**Why Was This Study Done?:**

Although epidemiological studies (investigations of the causes, distribution, and control of disease in population) show a correlation between antibiotic use and antibiotic resistance in populations, such studies cannot prove that antibiotic use actually causes antibiotic resistance. It could be that the people who use more antibiotics share other characteristics that increase their chance of developing antibiotic resistance (so-called “confounding”). A causal link between antibiotic use and the development of antibiotic resistance can only be established by doing a randomized controlled trial. In such trials, groups of individuals are chosen at random to avoid confounding, given different treatments, and outcomes in the different groups compared. Here, the researchers undertake a randomized clinical trial to assess whether macrolide resistance is higher in communities treated with azithromycin for trachoma than in untreated communities. Azithromycin—an erythromycin derivative—is used to treat common bacterial infections such as middle ear infections caused by *Streptococcus pneumoniae*. Trachoma—the world's leading infectious cause of blindness—is caused by *Chlamydia trachomatis*. The World Health Organization's trachoma elimination strategy includes annual azithromycin treatment of at-risk communities.

**What Did the Researchers Do and Find?:**

In this cluster-randomized trial (a study that randomly assigns groups of people rather than individuals to different treatments), 12 Ethiopian communities received mass azithromycin treatment of children aged 1–10 years old at 0, 3, 6, and 9 months, and 12 control communities received the antibiotic only at 12 months. The researchers took nasopharyngeal (nose and throat) swabs from randomly selected treated children at 0 and 12 months and from randomly selected control children at 12 months. They isolated *S. pneumoniae* from the swabs and tested the isolates for antibiotic susceptibility. 70%–80% of the children tested had *S. pneumoniae* in their nose or throat. In the treated group, 3.6% of monitored children were carrying azithromycin-resistant *S. pneumoniae* at 0 months, whereas 46.9% were doing so at 12 months—a statistically significant increase. Only 9.2% of the monitored children in the untreated group were carrying azithromycin-resistant *S. pneumoniae* at 12 months, a significantly lower prevalence than in the treated group. Importantly, there was no resistance to penicillin in any *S. pneumoniae* isolates obtained from the treated children at 0 or 12 months; one penicillin-resistant isolate was obtained from the control children.

**What Do These Findings Mean?:**

These findings indicate that macrolide resistance is higher in nasopharyngeal *S. pneumoniae* in communities receiving intensive azithromycin treatment than in untreated communities. Thus, they support the idea that frequent antibiotic use selects for antibiotic resistance in populations. Although the study was undertaken in Ethiopian communities with high rates of nasopharyngeal *S. pneumoniae* carriage, this finding is likely to be generalizable to other settings. Importantly, these findings have no bearing on current trachoma control activities, which use less frequent antibiotic treatments and are less likely to select for azithromycin resistance. The lack of any increase in penicillin resistance, which is usually the first-line therapy for *S. pneumoniae* infections, is also reassuring. However, although these findings suggest that the benefits of mass azithromycin treatment for trachoma outweigh any potential adverse affects, they nonetheless highlight the importance of continued monitoring for the secondary effects of mass antibiotic distributions.

**Additional Information:**

Please access these Web sites via the online version of this summary at http://dx.doi.org/10.1371/journal.pmed.1000377.

- The Bugs and Drugs website provides information about antibiotic resistance and links to other resources
- The US National Institute of Allergy and Infectious Diseases provides information on antimicrobial drug resistance and on diseases caused by S. pneumoniae (pneumococcal diseases)
- The US Centers for Disease Control and Prevention also have information on antibiotic resistance (in English and Spanish)
- The World Health Organization has information about the global threat of antimicrobial resistance and about trachoma (in several languages)
- More information about the trial described in this paper is available on ClinicalTrials.gov

## Introduction

Antibiotic selection pressure is thought to be an important mechanism of selecting for antibiotic resistance in populations [1]. High antibiotic use is correlated with antibiotic resistance in ecological studies [2]–[10], and cross-sectional, cohort, and case-control studies have confirmed these findings [11]–[13]. Although these studies suggest that population-level antibiotic pressure is associated with resistance, these study designs are subject to bias [14],[15]. A randomized controlled trial would provide the strongest evidence for a causal relationship between community antibiotic consumption and resistance.

Trachoma, caused by infection with ocular strains of *Chlamydia trachomatis*, is the leading infectious cause of blindness worldwide. The World Health Organization (WHO) endorses mass distributions of antibiotics as one component of an integrated trachoma control strategy. Mass antibiotic treatment clears chlamydial infection in both symptomatic and asymptomatic individuals, thus reducing the infectious reservoir of disease [16]. Most programs distribute community-wide azithromycin annually, though there is evidence that the most severely affected communities may require more frequent antibiotic distribution for trachoma elimination [17]–[19].

Communities receiving mass azithromycin treatments for trachoma are under intense antibiotic selection pressure. Although chlamydial resistance has not been reported [20],[21], nasopharyngeal pneumococcal resistance has been observed in uncontrolled studies after a single azithromycin treatment, and after repeated annual treatments [22]–[24]. Recently, we performed a population-based, cluster-randomized clinical trial of mass azithromycin for trachoma in Ethiopia [25]. In this study, entire communities were randomized either to intensive azithromycin treatments, or to no treatment, and monitored for trachoma. The trial also provided a unique opportunity to further characterize the community-level effects of antibiotic pressure on resistance. Here, we report nasopharyngeal *S. pneumoniae* resistance in children before and after frequent mass azithromycin treatments, and compare to untreated control communities.

## Methods

The study had approval from the Committee for Human Research of the University of California, San Francisco, Emory University, and the Ethiopian Science and Technology Commission. The study was carried out in accordance with the Declaration of Helsinki and overseen by a Data Safety and Monitoring Committee appointed by the National Institutes of Health-National Eye Institute.

### Setting

**Figure 1:**
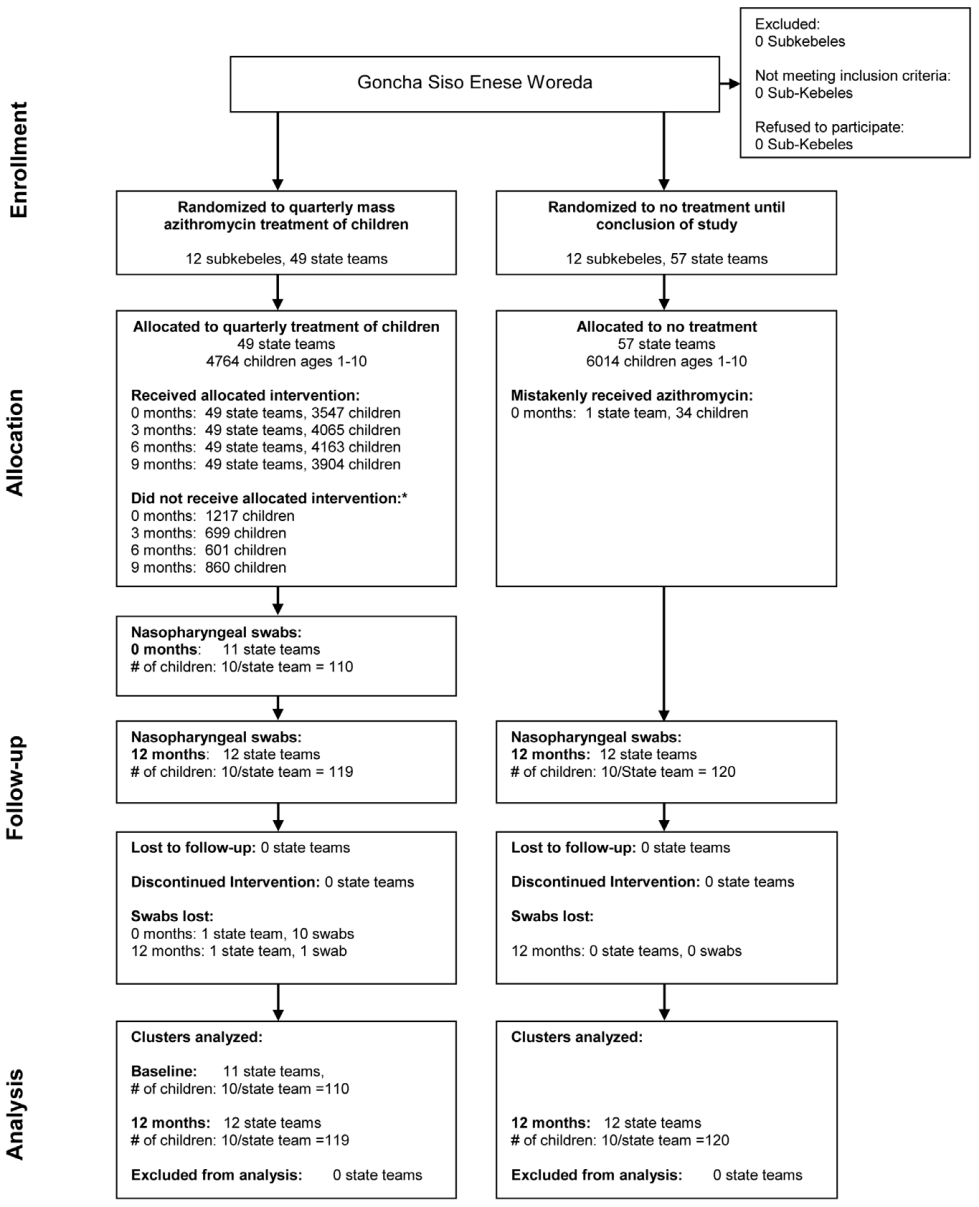
Trial profile. 24 subkebeles were randomized to mass treatment of children, or to a control group that received delayed treatment after the conclusion of the study. No sentinel communities were lost to follow-up, and none discontinued the intervention. All communities were included in the analyses at 12 mo. *Reasons for not receiving allocated intervention included absent, moved, or death.

This study consists of a prespecified analysis from a cluster-randomized clinical trial conducted between May 2006 and May 2007 in the Goncha Siso Enese *woreda* (district) of the Amhara zone of Ethiopia [25]. As part of the clinical trial, 12 *subkebeles* (administrative units) were randomized to receive quarterly azithromycin treatment of children ages 1–10 y at months 0, 3, 6, and 9. Twelve control subkebeles were randomized to treatment of the entire community at month 12. Subkebeles were randomly chosen from an area of 72 contiguous subkebeles. This area excluded the local town, where the prevalence of trachoma would likely be low [26], and inaccessible communities (defined as those greater than a 3-h walk from the furthest point available to a four-wheel drive vehicle). The socioeconomic and demographic characteristics of the included subkebeles were similar. The randomization sequence was generated by Kathryn Ray with the RANDOM and SORT functions in Excel (Version 2003) and concealed until assignment. Participant enrollment and treatment assignment was performed by BA. Each subkebele consisted of approximately four to six state teams (administrative subunits), termed “communities” for this report. All communities from the randomized subkebeles were treated identically to minimize contamination between study arms. One randomly chosen sentinel community was monitored for trachoma (results described elsewhere) [25]. In addition, nasopharyngeal *S. pneumoniae* antibiotic resistance was assessed in the sentinel communities, and is reported here (Figure 1; Texts S1 and S2).

### Intervention

In the children-treated arm, all children aged 1–10 y were offered one dose of directly observed oral azithromycin (20 mg/kg) every 3 mo for 1 y (at months 0, 3, 6, and 9). Treatments were offered to all children in the subkebele during a single antibiotic campaign lasting several days, and all subkebeles in the study were treated within several weeks of each other. In order to monitor for a secular trend, a delayed treatment arm (control arm) was enrolled at baseline, but not monitored until month 12, after which all individuals aged 1 y and older were offered azithromycin treatment. In both treatment groups, macrolide-allergic or self-reporting pregnant individuals eligible for treatment were offered a 6-wk course of twice-daily 1% tetracycline ointment. Antibiotic coverage was assessed by the antibiotic distributors against the baseline census. For ethical reasons, other than the baseline census, we collected no data from the control group until month 12 of the study.

### Outcome Participants

We collected nasopharyngeal samples from ten randomly selected children aged <10 y from each sentinel community in (1) the children-treated arm at baseline and month 12, and (2) the control arm at month 12 only. Swabs were always collected just before a mass azithromycin distribution; therefore, the swabs collected at month 12 in the children-treated arm were collected 3 mo after the most recent treatment. The random sample was redrawn at each visit, so children selected at baseline may or may not have been selected again at month 12. An alternative list of five additionally randomly selected children was available at each visit, to be used if any of the first ten randomly selected children had moved, died, or were traveling during the collection period. Verbal informed consent was obtained from parents or guardians for each child in the local language, Amharic.

### Sample Collection

Nasopharyngeal swabs were preserved and transported using skim milk-tryptone-glucose-glycerin medium, as previously described [27]. Samples were kept on ice in the field and then in a −20°C freezer, and subsequently shipped to the United States on ice. Samples arrived frozen, and were placed in a −80°C freezer for up to 6 mo until processed.

### Laboratory Studies

*S. pneumoniae* colonies were identified using selective media (incubated at 35°C in 5% CO_2_) and optochin and bile solubility testing. *S. pneumoniae* isolates were evaluated for antimicrobial susceptibilities using Etest strips (bioMérieux - AB Biodisk) placed on Mueller-Hinton agar plates with 5% defibrinated sheep blood, which were incubated at 35°C in 5% CO_2_ for 20–24 h before determination of minimum inhibitory concentrations (MICs). *S. pneumoniae* ATCC 49619 was used for quality control in each run. MIC values were determined from the FDA-approved package insert, using interpretation values for CO_2_ when provided. The following MICs were used to define resistance: azithromycin (CO_2_) (≥16 µg/ml), clindamycin (CO_2_) (>2 µg/ml), benzylpenicillin (≥2 µg/ml), and tetracycline (≥8 µg/ml). All susceptibility testing was performed by a technician masked to study arm and time point.

### Genotyping

All azithromycin-resistant isolates underwent genotypic analysis for the *mefA* gene (M phenotype, drug efflux) and *ermB* gene (MLS_B_ phenotype, ribosomal target modification). These two genes account for the vast majority of azithromycin resistance [28]–[31]. PCR was performed using oligonucleotide primers to amplify a 348-bp segment containing the *mefA* gene or a 639-bp segment containing the *ermB* gene element [32]. Positive controls for each primer pair and a negative control strain (*S. pneumoniae* ATCC 49619) were included with all runs.

### Statistical Analysis

Analyses were conducted at the community level. Antibiotic coverage was defined as the proportion of children aged ≤10 y who accepted treatment with azithromycin or tetracycline at each time point, as determined from the baseline census. Note that children aged under 1 y were not eligible for azithromycin treatment, but were included in the coverage calculations since they were monitored for resistance. *S. pneumoniae* carriage was defined as the proportion of nasopharyngeal samples from which *S. pneumoniae* was isolated. *S. pneumoniae* resistance was defined as the proportion of *S. pneumoniae* isolates that displayed antibiotic resistance. The mean prevalence of *S. pneumoniae* carriage and *S. pneumoniae* resistance in children aged <10 y was estimated from the 12 sentinel communities of the children-treated arm at baseline and 1 y, and from the control arm at 1 y. The average proportion of resistant isolates that tested positive for *ermB* and *mefA* determinants was calculated, using only those communities with resistant isolates. Bias-corrected bootstrapped 95% confidence intervals (CIs) were computed (10,000 repetitions). If there were no observations for a proportion, exact binomial one-sided 97.5% CIs were calculated, ignoring clustering. The prespecified primary outcome compared the prevalence of resistance between communities in the treated and control arms at month 12 (Wilcoxon rank sum test). An additional prespecified outcome was the comparison of the prevalence of resistance within communities in the treated arm comparing month 0 to month 12 (Wilcoxon signed-rank test). Several non-prespecified analyses were also conducted. We performed univariate mixed effects logistic regression on the population of children colonized with pneumococcus, with the presence of azithromycin resistance at 12 mo as the response variable, and the treatment arm as the explanatory variable, while clustering at the subkebele level. We calculated the prevalence of antibiotic resistance among all monitored children, regardless of whether pneumococcus was isolated. The intraclass correlation (ICC) for the children-treated arm at 12 mo was calculated using the *loneway* command in Stata. As an exploratory analysis, an r×c contingency table (2 rows, 12 columns) was constructed plotting the presence or absence of the *mefA* genetic determinant against sentinel community, and a Fisher exact test was performed to test whether *mefA*-positive isolates were evenly distributed among communities. An identical analysis was performed for *ermB*. Intention-to-treat analyses were performed for all statistical tests. The trial had 90% power to detect a 30% difference between the two groups, assuming 24 clusters randomized in a 1∶1 allocation ratio, a *S. pneumoniae* carriage rate of 80%, a type-I error rate of 0.05, an ICC of 0.05 (determined from a previous study [33]), and a 50% prevalence of azithromycin resistance in the treated group at 12 mo. All statistical analyses were performed with Stata version 10.0.

## Results

### Characteristics of Treatment Arms

The pretreatment characteristics of the children included in the two study arms were not significantly different (Table 1). Among children ages ≤10 y in the sentinel communities of the treated group, azithromycin coverage at months 0, 3, 6, and 9 was 72.8% (95% CI 67.6%–76.9%), 76.3% (72.2%–80.1%), 80.4% (77.9%–2.9%), and 78.2% (75.2%–80.7%), and tetracycline coverage was 1.5% (0.6%–2.9%), 1.5% (0.6%–2.7%), 2.5% (1.1%–4.5%), and 4.8% (3.5%–6.2%), respectively. In the control arm, 34 children were mistakenly treated in one subkebele at baseline, including 12 children from the sentinel community. This mistakenly treated control sentinel community was retained in the control group for all analyses.

**Table 1:** Pretreatment characteristics of children in the children-treated group and the control group.

Characteristic	Children-Treated	Control^a^
Population per community^b^	100.5 (80.1–120.9)	104.2 (92.7–115.7)
Age, y^b^	4.4 (4.3–4.5)	4.5 (4.3–4.6)
Female^b^	49.3% (46.6%–52.0%)	48.7% (47.1%–50.2%)
Clinically active trachoma^c^	69.0% (57.5%–80.5%)	70.0% (62.2%–77.6%)
Ocular chlamydia^d^	48.4% (42.9%–53.9%)	45.6% (36.7%–54.5%)

Estimates represent the mean, shown with 95% CIs in parentheses.

a Observations for the control group are, by design, from the 1-y time point.

b Demographic characteristics reported for children ages <10 y from all study communities (state teams).

c Defined as follicular trachomatous inflammation (TF) and/or intense trachomatous inflammation (TI) by the WHO simplified grading scale; reported for children ages 1–10 y in the sentinel communities [45].

d As detected by PCR; reported for children ages 1–10 y in the sentinel communities.

### *S. pneumoniae* Carriage and Antimicrobial Susceptibility of Isolates

We collected a random sample of ten nasopharyngeal swabs from each sentinel community in the children-treated group at baseline and at month 12, and from each sentinel community in the control group at month 12. In a single community in the treated arm, all baseline samples were destroyed during a flood, and one sample was lost at month 12. Baseline characteristics of the community with missing data were not significantly different from the other sentinel communities (unpublished data). *S. pneumoniae* was isolated from 76 of 110 nasopharyngeal samples collected from the treated arm before mass azithromycin treatments (mean prevalence of *S. pneumoniae* carriage 69.1% [95% CI 56.7%–81.7%]), and from 93 of 119 samples 12 mo after the baseline treatment (78.0% [68.0%–86.7%]) (Table 2). Data was collected from the control group at month 12 only; in this untreated group, *S. pneumoniae* was isolated in 98 of 120 nasopharyngeal samples (81.7% [95% CI 75.8%–89.2%]) (Table 2).

**Table 2:** Nasopharyngeal pneumococcal carriage and resistance in children aged <10 y in the children-treated group (pre- and post-treatment), and the untreated control group.

Carriage or Resistance	Azithromycin-Treated (*n* = Communities)			Control (*n* = Communities)		
	Baseline Pretreatment *n* = 11	Month 12 Post-treatment *n* = 12	*p*-Value^a^	Baseline (Not Sampled by Design)	Month 12 Untreated *n* = 12	*p*-Value^b^
*S. pneumoniae* carriage^c^	69.1% (56.7%–81.7%)	78.0% (68.0%–86.7%)	0.09	—	81.7% (75.8%–89.2%)	0.72
**Azithromycin resistance**						
Swabs^d^	3.6% (0.8%–8.9%)	46.9% (37.5%–57.5%)	0.003	—	9.2% (6.7%–13.3%)	<0.0001
Isolates^e^	6.3% (1.0%–15.7%)	62.3% (49.1%–75.4%)	0.003	—	11.6% (6.9%–17.1%)	0.0001
**Clindamycin resistance**						
Swabs^d^	1.5% (0%–6.3%)	13.3% (6.7%–23.3%)	0.02	—	3.3% (0.8%–8.3%)	0.10
Isolates^e^	1.5% (0%–6.1%)	16.9% (6.9%–27.9%)	0.02	—	3.9% (1.0%–8.6%)	0.10
**Penicillin resistance**						
Swabs^d^	0% (0%–3.3%)	0% (0%–3.1%)	—	—	0.8% (0%–4.2%)	0.32
Isolates^e^	0% (0%–4.7%)	0% (0%–3.9%)	—	—	1.0% (0%–5.2%)	0.32
**Tetracycline resistance**						
Swabs^d^	10.0% (4.5%–18.2%)	28.4% (19.4%–38.4%)	0.02	—	17.5% (11.7%–24.2%)	0.11
Isolates^e^	15.2% (5.6%–28.1%)	35.5% (24.7%–45.2%)	0.04	—	21.5% (13.9%–28.7%)	0.07

Estimates represent the mean of sentinel communities, shown with 95% confidence intervals in parentheses.

a Wilcoxon signed rank test, comparing pre- and post-treatment in the treated arm.

b Wilcoxon rank sum test, comparing post-treatment treated arm with untreated control arm.

c Proportion of nasopharyngeal samples from which *S. pneumoniae* was isolated.

d Proportion of swabbed children who were classified as resistant.

e Proportion of pneumococcal isolates that were classified as resistant.

The prevalence of antibiotic resistance among children aged <10 y is shown for the population of all swabbed children, and for the population of children from which pneumococcus was isolated (Table 2). Prior to treatment, three of the 11 sentinel communities in the treated group demonstrated azithromycin-resistant *S. pneumoniae* isolates, and a total of four of the 76 isolates were resistant (mean prevalence among pneumococcal isolates, 6.3% [95% CI 1.0%–15.7%]) (Table 2). After four azithromycin treatments within 1 y, azithromycin resistance was observed in all 12 communities, with 56 of 93 isolates demonstrating resistance (intraclass correlation [ICC]  = 0.11 [95% CI 0–0.29]; mean prevalence 62.3% [95% CI 49.1%–75.4%], *p* = 0.003 compared to baseline, prespecified analysis). In the control group at month 12, nine of 12 communities exhibited azithromycin-resistant strains, with 11 of the 98 isolates testing positive for resistance (mean prevalence 11.6% [95% CI 6.9%–17.1%], *p* = 0.0001 compared to children-treated group at 1 y, prespecified analysis). Children from communities treated with quarterly mass antibiotics were more likely to be colonized with macrolide-resistant pneumococcus compared to children from untreated communities; OR 13.2 (95% CI 5.5–31.9; non-prespecified analysis).

Significant increases in clindamycin and tetracycline resistance were detected after mass antibiotic distributions (Table 2). In the treated arm, clindamycin resistance increased from one resistant isolate before mass treatment (mean prevalence 1.5% [95% CI 0%–6.1%]) to 16 resistant isolates after four quarterly treatments (mean prevalence 16.9% [6.9%–27.9%], *p* = 0.02), though this level was not significantly higher than time-matched untreated controls (four resistant isolates, corresponding to 3.9% of isolates [95% CI 1.0%–8.6%], *p* = 0.10). Before treatment, children carried strains resistant to tetracycline more than any other antibiotic tested, with 11 resistant isolates at the baseline visit in the children-treated arm (15.2% of all isolates [95% CI 5.6%–28.1%]). By month 12, 34 tetracycline-resistant isolates were recovered in the treated group (mean prevalence 35.5% [95% CI 24.7%–45.2%], *p* = 0.04), though this was not significantly greater than that of time-matched untreated controls (21 resistant isolates; mean prevalence 21.5% [13.9%–28.7%], *p* = 0.07).

The only antibiotic tested that did not demonstrate the emergence of significant resistance was benzyl-penicillin. Penicillin resistance in the community was rare, with no resistant isolates observed in the children-treated group, either before or after mass antibiotic treatments. In the untreated control group, a single resistant isolate was identified, which corresponded to 1.0% (95% CI 0%–5.2%) of the population.

### Genotyping of Azithromycin-Resistant Isolates

The average proportion of azithromycin-resistant strains testing positive for the two most common genetic elements encoding resistance is shown in Table 3. All *ermB+* isolates, including 17 *mefA−/ermB+* isolates and 4 *mefA+/ermB+* isolates, also had high-level azithromycin resistance by Etest (MIC>256). Azithromycin resistance was moderate in the 49 *mefA+/ermB−* isolates (range 24 to >256; median  = 192). In comparison, the 196 susceptible isolates had azithromycin MICs ranging from 0.19 to 2 (median = 1).

**Table 3:** Genotypic characteristics of azithromycin-resistant isolates from children aged <10 y old in the treated group (pre- and post-treatment), and the untreated control group.

Genetic Determinant	Azithromycin Treated (*n* = Communities)			Control (*n* = Communities)		
	Baseline Pretreatment *n* = 11	Month 12 Post-treatment *n* = 12	*p*-Value^a^	Baseline (Not Sampled by Design)	Month 12 Untreated *n* = 12	*p*-Value^b^
*mefA+/ermB−*	66.7% (0%–100%)	72.8% (52.8%–89.5%)	0.32	—	66.7% (36.0%–100%)	0.91
*mefA−/ermB+*	33.3% (0%–100%)	19.4% (7.1%–34.4%)	0.32	—	33.3% (10.0%–72.7%)	0.81
*mefA+/ermB+*	—	6.6% (2.1%–13.9%)		—	—	
*mefA−/ermB−*	—	1.2% (0%–5.6%)		—	—	

Estimates represent the mean proportion of azithromycin-resistant isolates, shown with 95% CIs in parentheses.

a Wilcoxon signed rank test, comparing pre- and post-treatment in the treated arm.

b Wilcoxon rank sum test, comparing post-treatment treated arm with untreated control arm.

Four azithromycin-resistant isolates were detected at baseline in the children-treated group. The three isolates with *mefA* resistance at baseline came from two communities, and each of these communities demonstrated only *mefA* resistant strains at month 12. The *ermB* genetic determinant was seen in a separate community at baseline; at the 12-mo follow-up, this community demonstrated predominantly *ermB* resistance, but also a single isolate with both determinants. In seven of the 12 treated communities at 12 mo, all resistant isolates displayed the *mefA* genetic determinant (*p* = 0.09 for 2×12 contingency table). In contrast, there was only one community in the treated group at 1 y in which all isolates exhibited the *ermB* determinant (*p* = 0.01 for 2×12 contingency table). The prevalence of the *mefA* determinant in the children-treated group at month 12 did not differ from the prevalence of *mefA* in the children-treated group at baseline (*p* = 0.32), or from the control group at month 12 (*p* = 0.91).

## Discussion

This cluster-randomized clinical trial demonstrates that frequent antibiotic use selects for community-level antibiotic resistance. In communities randomized to four azithromycin treatments within 1 y, azithromycin resistance was observed in 47% of all swabbed children and 62% of children colonized with pneumococcus; this was significantly higher than untreated control communities, in which resistance was found in 9% of swabbed children and 12% of children colonized with pneumococcus. Genotype analyses were consistent with the widely accepted theory that antibiotic selection pressure increases community antibiotic resistance by reducing susceptible bacterial strains and allowing clonal expansion of existing resistant strains.

Numerous ecological, analytic, and interventional studies have suggested that population-level antibiotic pressure selects for antibiotic resistance [34]–[36]. However, it has been difficult to rule out the possibility of bias in many of these studies, since antibiotic use in a population is difficult to quantitate, resistance testing is rarely population based, and unmeasured confounders cannot be ruled out. The study design used here had several advantages that helped minimize bias. First, our knowledge of the degree of antibiotic use was extremely accurate. This study was conducted in a rural region in Ethiopia with infrequent background macrolide use. A large, known amount of oral azithromycin was distributed to treated communities, and treatment was directly observed. Second, the study was a randomized controlled trial, which greatly reduces the possibility of an association caused by unmeasured confounders. Third, this study was cluster randomized. Although a previous clinical trial showed antibiotic resistance in individuals using macrolides [36], the cluster randomization of this study allows analysis of community-level antibiotic resistance. Furthermore, the likelihood of contamination from surrounding communities was reduced by randomizing government districts (subkebeles), and treating all communities in the district identically. Finally, bias was reduced by performing population-based monitoring on a random sample of children.

Antibiotic selection pressure is thought to increase community antibiotic resistance by reducing susceptible bacterial strains and shifting the competitive balance in favor of existing resistant strains [1]. The distribution of the *mefA* and *ermB* genetic determinants in this study suggests that clonal expansion of resistant strains occurred. For example, the three communities with a specific genetic element at baseline demonstrated a greater prevalence of that same determinant after treatment. In addition, genes encoding resistance were often present as an “all or none” phenomenon in a particular community, suggesting the spread of existing genetic determinants, rather than development of new ones. However, given the wide CIs around the prevalence of *mefA* and *ermB*, these results should be interpreted with caution.

Although we did not follow communities after treatments were stopped, there is evidence to suggest that pneumococcal resistance is transient in areas with endemic trachoma. In an uncontrolled study of a single community in Australia, 35% (10/29) of treated children exhibited macrolide resistance 2 mo after a single dose of azithromycin, but only 6% (2/34) did so 6 mo later [22]. Although this community did not receive mass azithromycin—approximately half of children received azithromycin—the study nonetheless suggests that resistance fades after antibiotic selection pressure is removed. In other studies, macrolide resistance was observed in only 5% of children 6 mo after the last of two or three annual mass treatments [23],[24]. These findings were corroborated by a population-based study in Ethiopia, in which the prevalence of macrolide resistance decreased dramatically after cessation of mass azithromycin treatments—from 77% after the last of six biannual mass treatments, to 21% by 2 y after the final treatment [33].

In this community-randomized clinical trial, quarterly azithromycin treatment of children was clearly effective in reducing ocular chlamydia [25]. Here, however, we report that more frequent mass treatments also select for pneumococcal resistance. It is notable that even the periodic treatments of this study were sufficient to select for resistant strains, at least in this rural Ethiopian setting with presumably efficient interhost transmission. Fortunately, any negative impact is tempered by several factors. First, resistance to penicillins, which would serve as first-line therapy for *S. pneumoniae* infections and are widely available and used in the study area, was not detected. Second, macrolides are rarely used in the region, based upon a survey of pharmacies (BA, unpublished data). Third, azithromycin resistance appears to be transient in similar communities once mass treatments are stopped [22],[33]. Fourth, the clinical significance of azithromycin resistance is unclear. There have been no reports of increased invasive pneumococcal disease or increased mortality in areas treated with mass azithromycin. To the contrary, a concomitant trial found that mass azithromycin distributions may even reduce childhood mortality [37]. Finally, in this study, antibiotics were distributed every 3 mo—much more frequently than the annual treatments recommended by WHO guidelines. This study is quite different from previous studies, which have monitored pneumococcal resistance after much lower levels of antibiotic selection pressure. In particular, this study cannot be extrapolated to the case of repeated annual mass treatments, for which pneumococcal macrolide resistance has never been shown to exceed 5% by 6 mo after treatment [23],[24]. Likewise, this study cannot be generalized to the case of a single mass azithromycin distribution, for which other studies have found at most only a single isolate of pneumococcal macrolide resistance between 6–12 mo after a community-wide treatment [38],[39].

The beneficial effects of mass azithromycin treatments for trachoma are very clear. Mass azithromycin distributions for trachoma have been tremendously successful in reducing the prevalence of ocular strains of chlamydia, and may even result in the elimination of infection in some areas [18],[40]–[42]. These activities will be instrumental in reducing blindness due to trachoma. The adverse effects of mass treatments are much less certain. Although we show considerable nasopharyngeal macrolide resistance following frequent mass azithromycin in this study, there is good reason to think that the clinical impact of resistance is minimal, as discussed above. We believe that the known benefits of mass azithromycin treatments clearly outweigh any uncertain adverse effects, and that trachoma programs should continue to distribute mass azithromycin treatments.

This study has several limitations. We did not collect baseline nasopharyngeal samples in the control group, since we did not want to mislead participants, who might have construed swabbing as treatment. Note, however, that because treatment group was randomly assigned, baseline measurements are not necessary for between-group comparisons. We did not collect cultures from other sites to monitor for invasive pneumococcal diseases such as meningitis, pneumonia, or bacteremia. We do not have follow-up data for these communities. We have not performed a genetic analysis of pneumococcal strains, though we do plan on completing such an analysis in the future. This study was performed in Ethiopian communities with very high rates of pneumococcal carriage. Although this rate of pneumococcal carriage is the norm in much of Africa, it is higher than that seen in most industrialized countries [43],[44]. However, our findings are consistent with many studies conducted in developed countries [2]–[12], which suggests that the central finding of this study—that community level *S. pneumoniae* resistance increases with antibiotic use—is not specific to Ethiopia, but is generalizable to other settings.

This study demonstrates the importance of antibiotic selection pressure for community antibiotic resistance. Although we found a considerable amount of pneumococcal macrolide resistance in children treated with mass azithromycin treatments every 3 mo, this finding has no bearing on current trachoma control activities, which use less frequent antibiotic distributions, and which likely select for far less pneumococcal resistance [22]–[24],[38],[39]. Our findings nonetheless highlight the importance of continued monitoring for the secondary effects of mass oral antibiotic distributions.

## Supporting Information

Text S1 Trial protocol.
(0.52 MB DOC)

Text S2 CONSORT checklist.
(0.22 MB DOC)

## Abbreviations

CI: confidence interval
MIC: minimum inhibitory concentration
