# Supplementary material for: Antibiotic Selection Pressure and Macrolide Resistance in Nasopharyngeal Streptococcus pneumoniae: A Cluster-Randomized Clinical Trial
Source: PLoS Med. 2010 Dec 14;7(12):e1000377. doi: 10.1371/journal.pmed.1000377 (PMC3001893; doi:10.1371/journal.pmed.1000377)
Supplement: Text S1 — Trial protocol. (0.52 MB DOC) [file pmed.1000377.s001.doc]

*Version 5*

Eliminating Trachoma

With Repeat Mass Drug Treatment

# Manual of Operations and Procedures

# Francis. I. Proctor Foundation,

**The Carter Center**

### Investigators:

Teshome Gebre²

Paul Emerson, PhD²

Jenafir House, MPH, MSW^1^

Nicole Stoller, MPH^1^

Vicky Cevallos^1^

Zhaoxia Zhou^1^

Emmanuel Romero^1^

Travis C. Porco, PhD, MPH (Biostatistician)^1^

Bruce D. Gaynor, MD^1^

John P. Whitcher, MD, MPH^1^

Thomas M. Lietman, MD*^1^

*Principal Investigator

^1^Francis I. Proctor Foundation for Research in Ophthalmology, San Francisco, California, USA; ²The Carter Center, Atlanta, Georgia, USA & Ethiopia

### CONTENTS

### 1. Introduction

1.1 Specific Aims of the Study

1.2 Study Outcomes

1.2.1 Primary Outcome

1.3 Study Design

### 2. Background and Rationale

3. Organization and Policies

3.1 Collaborating Institutions

3.2 Duties & Responsibilities of Staff

3.2.1 Principal Investigator

3.2.2 Study Coordinators (Proctor Foundation)

3.2.3 Study Coordinator (The Carter Center, Ethiopia)

3.2.4 Co-Investigators (US & Ethiopia)

3.2.5 Microbiologist

3.2.6 Data Coordinator (The Carter Center, Ethiopia)

3.2.7 Database Specialist (Proctor Foundation)

- - 1. Laboratory Technician (Proctor Foundation)

3.2.9 Biostatistician/Data Analyst at Proctor

3.2.10 Treatment and Census Team: Local health extension workers from Ministry of Health, Ethiopia

3.2.11 Collection Team: Local health workers/nurses from Ethiopian Ministry of Health

3.3 Policy Matters

3.3.1 Protocol Revisions during the Trial

3.4 Presentations and Publications

3.4.1 Authorship Policy

3.5 Data Safety and Monitoring Committee

3.5.1 Stopping (& Restarting) Rules

4. Patient Flow

4.1 Eligibility Requirements

4.2 Randomization

4.3 Scheduling Visits

4.3.1 Baseline Visit

4.3.2 Treatment

4.3.3 Follow-up Visits

4.4 Adherence to Treatment

4.5 Adverse Outcomes and Patient Death

4.5.1 Adverse Outcomes

4.5.2 Patient Death

5. Examination and Procedures

5.1 Registering Participants for Examination

5.2 Gloving of the Examiner's Hands

5.3 Examination Positions

5.4 Everting the Upper Eyelid

5.5 Examining the Conjunctiva & Trachoma Grading

5.5.1 WHO Simplified Trachoma Grading Card

5.6 Field Specimen Collection Quality Control Measures

5.7 Examination Station and Diagram

6. Microbiology Laboratory Procedures for PCR

6.1 Specimen Collection for Microbiological Tests (Principal)

6.2 Materials

6.2.1 Swabs

6.2.2. Sample Tubes

6.2.3 Cooler Bags with Frozen Ice Packs

6.2.4 -20°C Freezer

6.2.5 Latex Gloves

- 1. Training of Examiners
     1. Pre-Monitoring Visit Training
     2. In-Country Training

6.4 Specimen Collection

6.4.1 Protocol for swabbing of the conjunctiva

6.4.2 PCR Control Swabs

6.4.3 Protocol for tubing and handling of samples

6.5 Transport and Storage of Samples

6.5.1 Materials for shipping

6.5.2 Packing the samples

6.6 Methods

6.6.1 Description of the Roche COBAS AMPLICOR™ CT/NG Assay

6.6.2 Processing of Samples for PCR

6.6.3 Procedure for Masking Samples

6.6.4 Procedure for Pooling Samples

6.7 Quality Control

6.8 Laboratory Results Reporting

7. Macrolide Resistance Testing

7.1 Specimen Collection for Macrolide Resistance Testing

7.2 Materials for NP Collection

7.2.1 Nasopharyngeal Swabs

7.2.2 Nasopharyngeal Sample Tubes

7.2.3 Cooler Bags with Frozen Ice Packs

7.2.4 -20°C Freezer.

7.2.5 Latex Gloves

7.3 Training of Examiners

7.4 Nasopharyngeal Specimen Collection

7.4.1 Protocol for Nasopharyngeal Sample Collection

7.5 Transport and Storage of Samples

7.6 Methods

7.6.1 Procedure for Masking Samples

7.6.2 Quality Control

7.6.3 Laboratory Results Reporting

8. Clinical Photography

8.1 Photography Protocol

8.2 Grading of Clinical Photographs

9. Study Medication

9.1 Study Medication Description (from Pfizer, Inc.)

9.2 Dosage Information

9.3 Adverse Reactions/Side Effects

9.4 Treatment Costs

9.5 Alternate Therapies (Control Villages)

9.5.1 Tetracycline Ophthalmic Ointment

9.6 Treatment/Monitoring Schedule

9.7 Medication Procurement/Donation

9.8 Study Medication Storage and Accountability

9.9 Medication Quality Control

9.10 Checking Antibiotic Coverage

### 10. Protection of Human Subjects

10.1 Internal Review Board Approval

10.1.1 UCSF Committee on Human Research

10.1.2 Ethiopian Science and Technology Commission

10.2 Informed Consent

10.3 Adequacy of Protection Against Risk

10.4 Inclusion of Pregnant Women & Children

10.5 Compensation to Participants

11. Data Collection and Management

11.1 Census administration

11.1.1 Form Description

11.1.2 Filling the Census Form

11.2 Field Data Collection

11.2.1 Registration Form

11.2.2 Field Data Form

11.3 Data Forms Management

11.3.1 Data Editing

11.3.2 Data Entry and Quality Control

11.3.3 Data Entry Errors

11.3.4 Data Consistency & Validity

11.4 Data Security

11.5 Data Storage

11.6 Data Analysis

11.7 Data Management, Security and Quality Assurance

12. Statistical Analyses

12.1 Calculation of Sample Size & Power Analysis

12.2 Inter-observer Variability

13. Appendix

13.1 Patient Verbal Consent Script

13.2 Census/Registration Form (The Carter Center)

13.3 Field Data Form (Swabbing)

14. References

**1.** **Introduction (taken from NIH Research Proposal U10EY016214)**

In this proposal we test the hypothesis that infectious trachoma can be eliminated from severely affected communities with repeated azithromycin distributions. Mass antimicrobial administrations have been used to combat parasitic diseases such as onchocerciasis, filariasis, and malaria with varying success.^1-3^ They have also been proposed for a variety of bacterial diseases, including syphilis and sexually transmitted Chlamydia.^4-6^ The World Health Organization ^7^ has initiated a comprehensive program to eliminate a bacterial disease, blinding trachoma, in large part based on the mass treatment of entire trachoma-endemic communities with oral azithromycin.^8^ There is little reason to believe that ocular chlamydia, the causative agent of trachoma, can be eliminated from a community with a single mass treatment—repeat treatment will almost certainly be necessary. Currently we do not know whether repeat treatment can eliminate infection. The common wisdom is that it cannot. In fact, the WHO has set a more conservative target of just reducing infection to a level where resulting blindness will not be a “public health concern”. Mathematical models suggest that complete elimination is theoretically possible, but only under certain conditions.^9^ Our preliminary results in a hyper-endemic area of Ethiopia suggest that these conditions may be present, however this will need to be tested empirically. Models also imply that children form a core group for the transmission of trachoma—if infection can be eliminated in this age-group then it would presumably fade away in adults, whose treatment now requires a substantial portion of a program’s resources. We have demonstrated that treating children alone can be successful in an area with a modest amount of trachoma. This strategy needs to be tested in a hyper-endemic area such as Ethiopia.

A bacterial disease has never been eliminated by a mass antibiotic program. If ocular chlamydial infection can be eliminated by repeat antibiotic treatment of only a subgroup of the population, then this would not only provide a rationale for trachoma programs, but also may encourage the use of similar strategies against a variety of bacterial diseases.

**1.1 Specific Aims of the Study**

**Specific Aim 1. To determine whether mass treatments can eliminate ocular chlamydia from hyper-endemic communities.** Will biannual repeat treatment be sufficient to reduce ocular infection in a community to zero? We will compare the prevalence of ocular chlamydial infection, and the number of communities in which infection has been eliminated, between study arms which have received treatment annually or biannually**.**

**Specific Aim 2. To determine whether children form a core group for the transmission of trachoma.** Can treatment targeted to children alone eventually eliminate infection in the entire community? We will monitor the effect of treating children alone on infection levels in the whole community by observing the prevalence of ocular chlamydial infection in a random sample of treated children as well as the older population who are not treated (indirect, herd effect of treatments targeted at children).

**Specific Aim 3. To determine whether latrine construction prevents the return of infection into a community after mass treatment.**  Can intensive latrine construction prevent trachoma from returning to treated areas by decreasing *Musca sorbens* population, a possible vector for trachoma? We will test antibiotics in the setting of non-antibiotic measures. This arm will be treated once with antibiotics at baseline and compared to an arm that’s treated once without intensive latrine construction.

**Specific Aim 4. To determine the effect of mass azithromycin treatments on antibiotic resistance in *Chlamydia trachomatis* and *Streptococcus pneumoniae*.**  We will study the impact of community-wide mass distribution of antibiotics on the emergence of macrolide resistance in *Chlamydia trachomatis* and *Streptococcus pneumoniae*, a common respiratory pathogen especially among children. Infant mortality will be monitored to study the health impact of mass antibiotic treatments.

**1.2 Study Outcomes**

**1.2.1 Main outcome measure**

Ocular chlamydial infection as detected by DNA amplification and polymerase chain reaction

**1.3 Study Design**

This study will be conducted as a longitudinal observational study. A total of 72 sub-kebeles will be randomly selected from 105 sub-kebeles in Goncha Siso Enese woreda (district) in East Gojam Zone, Amhara Regional State, Ethiopia and each of 12 sub-kebeles will be randomly assigned to one of the following 6 arms, corresponding to the Specific Aims (SA):

SA 1: a) Whole population treated with azithromycin annually

b) Whole population treated with azithromycin twice yearly

SA 2: c) Only children ≤10 treated four times in one year

d) Untreated, enrolled at twelve-months

SA 3: f) Whole population treated once only at baseline

g) Whole population treated once only at baseline plus latrines

All *state teams* (somewhat equivalent to a small village), made up of ~50 households (approximately 400 people, 120 children ≤9 years), in each study sub-kebele will be treated according to the Specific Aim to which their sub-kebele is assigned. The remaining, non-study sub-kebeles in Goncha Woreda and their state-teams will be treated according to The Carter Center (TCC) and Ministry of Health trachoma program.

Primary outcomes will be measured in one randomly chosen sentinel state-team for each sub-kebele (randomization unit) chosen for the study. Even though primary outcomes are measured in only one state-team per sub-kebele, the rest of state-teams in the same sub-kebele will be treated in the same manner to prevent the “contaminating” reintroduction of ocular infection from adjacent state-teams with a different treatment plan.

For baseline and follow-up surveys, a stratified random sample from two age groups will be chosen: 1) 60 study participants age 9 and younger and 2) 60 study participants aged 10 years and above. Clinical examination will be performed and conjunctival swabs will be taken from all study participants. In study arms F and G, only 60 study participants age 0 to ≤9 will be examined and receive conjunctival swabbing. For study arms C and D, nasopharyngeal swabs will be collected from 10 randomly selected children among the 60 participants age 0 to ≤ 9 who were recruited for conjunctival swabbing. Azithromycin will then be distributed accordingly. Mortality will be monitored in 60 out of 72 sub-kebeles (Arms A, B, C and D) for this study

If a state-team has no infection identified in the random sample of participants for two consecutive visits. Control sub-kebeles for arm D were not previously treated for trachoma and will be randomly selected for monitoring of ocular chlamydial infection. After monitoring, the sub-kebeles will exit the study at 12 months, and be treated as per the national Ethiopian Trachoma Control program.

### 2. Background and Rationale (taken from NIH Research Proposal U10EY016214)

**Mass drug administrations.** Mass antimicrobial treatment strategies have been used in several eradication campaigns and have been contemplated for many others. They have proven to be effective against some parasitic diseases (e.g., onchocerciasis and filariasis), but at times have not lived up to expectations (e.g., malaria).^1-3^ Various forms of mass treatment have been used for bacterial diseases, including sexually transmitted chlamydia and syphilis.^4, 6^ The World Health Organization ^7^ and their partners are now using repeated mass azithromycin administrations to control the ocular strains of chlamydia that cause trachoma, the world’s leading cause of infectious blindness.^10^ Unlike the eradication of a virus with vaccination (e.g., smallpox), the eradication of a bacteria with antibiotics may *not* be a realistic goal.^4^ The trachoma control program will be an interesting test of this.

**Trachoma control.** Trachoma is one of the leading causes of visual loss, accounting for 10-15% of world blindness.^10^ Although it disappeared long ago from western Europe and the United States, trachoma is still as endemic as ever in some parts of Africa, the middle east, Australia, and Southeast Asia.^8^ Ocular strains of *Chlamydia trachomatis* cause repeated episodes of conjunctivitis in children. In teenagers and adults, the disease progresses through a cascade of conjunctival scarring, in-turned eyelids (entropion), lashes touching the eye (trichiasis), and finally blinding corneal ulceration from bacterial or fungal infection of the abraded cornea.^11, 12^ The World Health Organization ^7^, in conjunction with non-governmental organizations, national-health services, and Pfizer Inc., recently began implementing a program designed to eliminate blinding trachoma.^8^ The WHO’s GET 2020 program (Global Elimination of Trachoma by the year 2020) has adopted a comprehensive set of control measures for trachoma-endemic areas summarized as the S.A.F.E. strategy - ^13^

Surgery for in-turned eyelids and lashes (entropion and trichiasis)

Antibiotics for infectious trachoma

Facial cleanliness to reduce transmission

Environmental improvements (clean water, fly control, etc.)

It is anticipated that the antibiotic component (in particular, single-dose oral azithromycin donated by Pfizer Inc.) will provide public health workers with the ability to prevent the transmission and recurrence of infectious trachoma before the onset of scarring and blindness. Most cases of ocular chlamydia are not symptomatic, so children cannot be expected to present for antibiotic treatment.^14^ Even if individual infections could be found, treated children are almost inevitably re-infected unless infection in the rest of the community is also addressed.^15^ Thus trachoma can only be reduced successfully through an extensive public health campaign that targets whole communities.

**Mass azithromycin distributions for trachoma.** Topical tetracycline was for a long time the recommended treatment for active trachoma, but compliance to the regimen of two applications per day for 6 weeks is extremely poor, even under study conditions. Azithromycin is an azalide antibiotic, closely related to macrolides such as erythromycin. It has properties that make it an ideal treatment for chlamydia: high efficacy, intracellular accumulation, and a long tissue half-life.^16-21^ A single dose of azithromycin is 92-98% effective in the elimination of both genital and ocular chlamydia from an individual, making compliance a non-issue and leading to the hope that this would be the *magic bullet* for trachoma.^22-32^ Mass distribution of azithromycin has been found to reduce the prevalence of clinically active trachoma in Morocco,^33^ Nepal,^34^ and Australia.^35^ Three doses (1 per week for 3 weeks) of azithromycin given to all members of a village drastically reduced the prevalence of chlamydial infection in Tanzania, The Gambia, and Egypt.^36^ Although very little is known concerning the long-term effect of mass treatments on chlamydia infection, results of repeat treatments are encouraging.^37^  However, it is unclear if periodic treatments can eventually eliminate infection, or if infection will just return to its baseline level after treatment is stopped. *Without answers to these questions, we currently do not have a proven rationale for long term mass antibiotic administrations.*

### Azithromycin safety: long-term use in children. Azithromycin has been safely used for acute bacterial infections in the pediatric population for over a decade. Recently, long-term, frequent-dosing of azithromycin in children has been investigated. It has been found to be effective for prophylaxing against bacterial pneumonia in children with cystic fibrosis,^38^ for preventing recurrent acute otitis media in children,^39^ and for treating *Mycobacterium* *avium*-complex infection in adult HIV patients.^40^ Studies for these indications have confirmed azithromycin’s safety profile in long-term use—each required far more frequent dosing of azithromycin than necessary for this study. The majority of side effects seen were minor gastro-intestinal symptoms.

**Emerging drug resistance.** Any campaign that includes mass distribution of antimicrobial agents must be concerned with adverse effects of the medicine, including the emergence of drug-resistant organisms. Mass distribution of oral sulfonamides to combat trachoma in northern Africa and the southwest United States in the 1950s and early 1960s was eventually halted, in part because of an unacceptable rate of side effects (most notably Stevens-Johnson Syndrome).^41^ The worldwide anti-malarial campaigns of the 1960s and 1970s never achieved their most optimistic goals at least partly because of emerging resistance.^3^ Chlamydial resistance can clearly be induced *in vitro* to many antibiotics, including sulfonamides, penicillins, rifampin, and fluoroquinolones.^42-44^ Clinical resistance has been described to sulfonamides, tetracycline,^45-47^ fluoroquinolones,^48^ and even macrolides (resistance to macrolides implies resistance to the closely related azalide azithromycin).^16, 49, 50^ These isolates tend to offer only heterotypic resistance, with only ~1% of any culture exhibiting resistance.^43^ The paucity of resistance reports implies that chlamydial resistance to azithromycin may be extraordinarily rare, but it should be kept in mind that antibiotic susceptibility in chlamydia is difficult to measure and rarely performed.^51^

On the other hand, *Streptococcus pneumoniae*, a common respiratory pathogen, frequently acquires resistance to macrolides. This organism colonizes 60-90% of children that are targeted for treatment in some trachoma-endemic areas.^52-55^ More than 97% of pneumococcal resistance to macrolides is mediated by either methylation of 23S rRNA (*ermAM*) or by drug efflux (*mefE*).^56-60^ A few studies, in Australia^53^ and Nepal,^54, 61, 62^ have assessed the impact of azithromycin distributions on pneumococcal resistance.  Leach et al. followed a cohort of children who had received azithromycin in a trachoma-endemic area of Australia (Figure 1).^53^ They found that azithromycin dramatically reduced the prevalence of sensitive pneumococcus (black curve). Some resistance to macrolides was seen at baseline, which was amplified after treatment (gray curve) but gradually disappeared as sensitive strains of pneumococcus repopulated the children over the subsequent 6 months. This study was limited by numbers (only 1 village, and only 39 children at the final, most important visit), and by lack of follow-up at 12 months, which is when a village would typically be retreated with antibiotic. Our group has monitored multiple villages after up to 3 treatments, and found only a modest amount of azithromycin resistance in pneumococcus.

**Figure 1**

Although investigators agree that no current data suggest mass azithromycin treatment has detrimental effects on the community, it will be important to monitor for resistance after multiple treatments, since the macrolide antibiotic pressure distributed in a trachoma program may exceed that in western populations.

**Children as a core group for transmission of trachoma.** In epidemiologic terms, a core group has been described as highly active transmitters of an infectious disease.^63, 64^ The criteria necessary to be a core group have been specified more precisely: if preventing transmission of infection in a subset of individuals does not allow infection to persist in the rest of the community, then this subset is a core group.^40^ There is evidence that children are particularly important for the transmission of trachoma, including:

1) Clinical activity and infection is far more common in children than in adults.^36, 65^

2) Infectious loads are higher in young children, as shown by direct fluorescent antibody testing (personal communication, Chandler Dawson) and recently confirmed by quantitative PCR.^66^

3) Chlamydial infection is of shorter duration in adults.^67-69^

4) Children are thought to have more contacts per day with others in the community than adults have.^70, 71^

5) A mathematical model suggests that treatment of just children several times per year should progressively reduce infection in the entire community.^9^ Even though untreated adults can occasionally re-infect children between treatments, if the children are treated frequently enough (e.g. every 2 or 3 months), then infection still eventually disappears in the entire community.^9^

**Non-antibiotic measures to control trachoma.** There is little doubt that trachoma disappeared in areas such as Western Europe due to socioeconomic improvements, even before the widespread use of antibiotics.^72, 73^ Likewise, many hope that adjunctive programs, such as the Facial cleanliness and Environmental improvement portions of the SAFE strategy, may help reduce infection.^8^ *However, despite the common wisdom, there is little available evidence that any particular non-antibiotic intervention can reduce the prevalence of chlamydial infection in a community.*^74^

**Facial cleanliness.** There is compelling anecdotal and circumstantial evidence that a clean face is associated with a lower risk of active trachoma.^72, 75, 76^ Active trachoma has been associated with poor water supply and dirty faces.^77^ However, evidence that face washing has a *causative* effect on the reduction of infection is marginal, with many studies lacking appropriate controls or masked outcomes.^74^ One well-designed interventional facial cleanliness study found that an intensive facial cleanliness program added *no* statistical benefit to the reduction of clinically active trachoma. Figure 2 above displays the average clinical activity in children after community-wide tetracycline ointment, with or without a face-washing program—the black line represents the average of 3 villages that received tetracycline, and the gray line, the average of 3 villages that received tetracycline *plus* the intensive face-washing program.^78^ As Figure 2 demonstrates, it is difficult to attribute a large additional effect to face washing.^79^ This may be because trials such as this have relied on clinical activity as an outcome and chlamydia infection itself may be a more sensitive indicator, or because the full effect of such programs is not realized after only 1 year. Or, it may be because face washing indeed has only a marginal effect on the prevalence of ocular chlamydial infection.

**Figure 2**

**Fly control.** There is circumstantial evidence that the face fly (*Musca sorbens*) participates in the transmission of trachoma.^80^ Active trachoma tends to be higher in seasons with higher fly density.^74^ On one occasion, fluorescein dye was thought to spread from one child to another via flies.^81^ Although it has been difficult to demonstrate the presence of chlamydial DNA on flies in hypoendemic areas,^82^ we recently found evidence of *C.trachomatis* DNA on 15% of flies from a hyperendemic village in Ethiopia.^83^ One interventional pilot study offers some intriguing results.^80^ Active trachoma was reduced more in two villages with an intensive (and unsustainable) fly control program than in two nearby villages without fly control.^84^ This is quite encouraging, although the observations were unmasked, and it is difficult to compare such small numbers of villages statistically.^85^ A well-designed fly control study involving multiple villages has recently taken place in the Gambia to try and address this important issue.^82^

***Chlamydia trachomatis*.** Chlamydiae are bacteria that are obligate intracellular parasites, unable to synthesize their own ATP. Strains of *C.trachomatis* are the cause of trachoma (Serotypes A-C), non-gonococcal urethritis (Serotypes D-K and occasionally sub-serotype Ba), and lymphogranuloma venereum (Serotypes L1-3). Ocular infections with genital strains (D-K) occasionally do occur, but are usually associated with genital disease, and are not commonly found in trachoma surveys.^86-88^ Rarely, ocular infections with Serotypes L1-3 have been reported. More recently, Genotypes have been used to describe the variation of chlamydial strains, even within a Serotype. The major outer membrane protein of the chlamydial elementary body has a great deal of variation, presumably to help evade the human host immune response. Because of its diversity, the gene for this protein (*OMP1*) has been used as a biomarker in molecular epidemiological studies.^87-94^

Humans are the definitive host for the ocular strains of *C.trachomatis*. The life cycle of chlamydia consists of an infectious elementary body stage that is non-replicating and not vulnerable to antibiotics, and an intracellular reticulate body phase where replication occurs. The length of this life cycle is about 72 hours. Thus antibiotics ideally must be present for a long period of time and accumulate intracellularly.

**Group-randomized trials.** Trachoma control is directed at a community, not just an individual. Therefore, clinical trials must randomize treatment strategies to the community (whether neighborhood, village, district, etc.); the analysis of these trials must take this into account.^85^ Many trachoma studies, including several of the most frequently cited, have randomized treatment at the village level but then analyzed results at the level of the individual, running the danger of falsely narrowing confidence intervals and inflating statistical significance.^36, 53, 78, 84^ The problem is that infections in 2 individuals in the same village are *not* independent events, and should not be analyzed as such—if one person is infected, his housemates and fellow villagers are also more likely to be infected.^95^ Even perfect matching of villages would not eliminate this effect, as the prevalence of infection in different communities varies stochastically. Thus, more villages, not more people within a village, are required for greater study power—findings from 1 or 2 villages are not useful statistically and can be misleading.

**Figure 3**

Figure 3 above illustrates how the power of a comparative study depends on the number of samples per village and the number of villages per treatment arm, in a setting where the intra-village correlation is 0.036.^85^ Note that doubling the number of villages adds far more power than doubling the number of samples per village, and that a study gains little power by sampling more than 50-75 individuals per village.

**Mathematical models of epidemics.** Mathematical models have been used to analyze the course of epidemics and the effect of public health measures, since the renowned Swiss mathematician Daniel Bernoulli justified the widespread use of smallpox vaccination with a model presented at the Royal Academy of Paris in 1760.^96, 97^ Epidemic models can take many forms, but one commonly used approach has been to divide a population into susceptibles and infectives.^70^ The number of infectives increases as susceptibles are infected by contact with infectious cases. As these infectious cases recover, they may again become susceptible or may become immune, depending on the particular disease being modeled. This process of infection and recovery has been modeled as a rate between the states with continuous time (differential equations), as a rate with discrete time (pseudo-Markov models, matrix algebra models), and as a chance event (stochastic models).^98^ By increasing the complexity of the model, age-structure,^9, 70^ preferential mixing within a household,^71, 99, 100^ strain variation,^101, 102^ partial immunity,^103^ and other details can be included. Models of ocular chlamydia transmission have been constructed by our group and others.^9, 104, 105^

**Pooling of chlamydial tests.** The clinical exam has limited accuracy in diagnosing chlamydial infections, particularly after treatment.^34, 36, 106-108^ DNA amplification tests (such as the polymerase chain reaction, PCR, and the ligase chain reaction, LCR) have proven to be extremely sensitive and specific in the detection of chlamydial sexually transmitted diseases.^109, 110^ Unfortunately, these tests are relatively expensive and are difficult to process in the areas with endemic trachoma. Programs are forced to use the clinical exam to determine who is most likely to be infected and thus most likely to benefit from antibiotics.

To reduce costs, pooling of chlamydia samples for DNA amplification testing is now often performed when monitoring sexually transmitted diseases; only samples coming from a positive pool need to be tested independently.^111-122^ When individual results are needed, savings are typically on the order of two-fold, depending on the prevalence in the population and how many samples are pooled into one test.^123^ If we do not need to know which individuals are infected but only the prevalence of infection in a community, then individual retesting of positive pools is NOT necessary, and even *greater efficiency* can be appreciated (~5-fold).^113, 121, 124^ The prevalence of infection can be estimated from the percentage of pooled specimens that are positive, using the simple formula:^111^

**Equation 1:** *Population Prevalence* = 1 – (1 – *Pooled Prevalence*),^1/n^

where *n* is the number of specimens placed into a pool.^111^ The confidence interval for the estimate of the population prevalence can be determined in a straightforward manner.^125^ Maximum likelihood methods can also be used to estimate the number of positive tests in a village from pooled results. Note that pooling allows longitudinal following of village prevalences, but not of individuals. Longitudinal analysis of individuals will not be used in this study, to avoid a cohort effect of children ‘aging-out’ of the higher risk groups over the course of several years.

**Goals for trachoma control.** We may not have to completely eradicate infection to prevent blindness due to trachoma. Ophthalmologists know that a single episode of genitally transmitted *C.trachomatis* eye infection (adult inclusion conjunctivitis) will typically not lead to scarring.^12^ Similarly, many suspect that less frequent or less severe episodes of infectious trachoma in children will never lead to cicatricial disease, and that only repeated, severe disease will lead to blindness.^126^ Many feel that trachoma is a disease where most morbidity can be eliminated even without eradicating the causative agent. Thus *elimination* may not be required to reduce blindness to an acceptable level.^4^ In fact, currently the WHO’s goal for trachoma programs is neither *eradication* nor true *elimination*, but the more conservative target of “elimination of trachoma as a public health concern” (essentially a euphemism for *control*, see Table 1). This is defined as less than 5% clinical activity in children. If reduction of ocular strains of chlamydia to zero locally (true elimination) is feasible with antibiotics alone, then this would provide a rationale for the trachoma program even without adjunctive measures—infection would presumably not return even after antibiotic treatments were discontinued.

|  | **Definition^4^** | **Example** |
| --- | --- | --- |
| **Eradication** | Reduction of infection incidence to zero worldwide | Smallpox |
| **Elimination** | Reduction of infection incidence to zero locally | Polio, Tetanus, Guinea Worm |
| **Elimination as a public health concern** | Disease specific: for example, reduction of the incidence of tuberculosis to <10/100,000/year or reduction of the prevalence of Leprosy to <1/10,000 | Tuberculosis, Leprosy |
| **Control** | Reduction of disease incidence to an acceptable level, requiring continued intervention | Cholera |

**Table 1**

**Significance.** The current rationale of the trachoma control program is to reduce chlamydial infection to a low level with antibiotics and then prevent it from coming back with better hygiene and fly control. While antibiotics can reduce the prevalence, it is not clear whether repeat mass treatments can eliminate infection or not. The common wisdom is that they cannot. There is a large difference between eliminating infection and just controlling it by reducing it to a low level. With elimination, infection will not return even after treatment is discontinued. If a low level of infection persists, then continued intervention is necessary. Continued long term intervention with antibiotics brings up issues of drug resistance and sustainability that have yet to be addressed.

The development of a proven long term rationale for mass azithromycin treatment is *essential* if the control program is to reach its goal of eliminating blinding trachoma from even the most hyper-endemic areas by the year 2020. Much is riding on the success of the trachoma elimination program—not only is trachoma one of the leading causes of blindness worldwide, but the strategy for elimination may have implications for other diseases. A bacterial disease has never been eliminated by mass antibiotic distributions. If ocular chlamydial infection can be eliminated by repeat antibiotic treatment, and if it can be done by targeting only a core group of the population, then this may provide a valuable strategy against a variety of bacterial diseases.

**3. Organization and Policies**

The Carter Center and the Proctor Foundation will jointly implement field research. The Carter Center will be responsible for organizing field operations and for liaising with all parties within Ethiopia, including the Ministry of Health, for the implementation of this research. The Proctor Foundation will be responsible for providing the necessary technical assistance and coordinating with The Carter Center, both in the field and at headquarters. The Carter Center and Proctor will abide by the Memorandum of Understanding (MOU), which includes mutually agreeable language specifying management roles and responsibilities, reporting, principal and co-investigators, and attribution of data and research. It was drafted and signed by the organization representatives from both collaborating parties.

**3.1 Collaborating Institutions**

**Francis I. Proctor Foundation**

The Proctor Foundation is an organized research unit at the University of California, San Francisco. The Foundation has a 56 year history of research in ocular infectious and inflammatory diseases and runs one of the leading corneal fellowship training programs in the United States. Proctor Foundation Faculty has been involved in prevention of blindness research in developing countries since the foundation’s inception. The impetus for establishing the foundation in 1947 was to eradicate trachoma in the American Southwest and in other parts of the world.

From this initial vision we have expanded our research efforts to include the other major causes of blindness worldwide, with a continuing emphasis on infectious and inflammatory eye diseases. Dr. Thomas Lietman, Principal Investigator of the study at Proctor Foundation, will be assisted by numerous co-investigators, two study coordinators, a microbiologist, a data management specialist, laboratory PCR processing staff, and a biostatistician at UCSF.

**The Carter Center – Ethiopia**

In October 2000, The Carter Center, with funding from Lions-Carter Center SightFirst Initiative, accepted the Ethiopian government's invitation to work on controlling trachoma in the Amhara region. When the program began in Amhara in 2000, trachoma was highly endemic among children less than 10 years old and adult women. Studies reported that approximately 88 percent of all children aged 1-10 years had active trachoma and an estimated 20 percent of adult women less than 40 years old were infected.

This baseline study further encouraged the program to conduct a needs assessment, which included interviews with schoolteachers. Studies revealed that most schoolteachers had minimal knowledge of the disease or its prevention. Teachers expressed interest in learning more and requested health education materials and structured guides for teaching trachoma prevention in the classroom.

The invaluable feedback led to discussions between The Carter Center and the Amhara Regional Health and Education bureaus. A working group developed a trachoma prevention curriculum and corresponding health education materials to cover all components of the SAFE strategy with an emphasis on "facial cleanliness" and "environmental sanitation." Each lesson encouraged students to identify other problems related to hygiene in their own communities and come up with their own solutions.

In addition to promoting school health education, the program continues ongoing health education promoting personal and environmental hygiene within the community. These activities target especially women and children.

To improve environmental sanitation, the program is promoting household latrine construction and use. From 2003 to June 2004, more than 70,000 household latrines were built in the Amhara region.

The Carter Center also worked with partner nongovernmental organizations to advocate for donation of Zithromax® from Pfizer Inc. As a result, in 2003, the program started mass treatment with azithromycin in trachoma-endemic communities. In 2004, a total of 625,422 people at risk for trachoma received treatment with azithromycin.

The Carter Center, Ethiopia Country Representative is Mr. Teshome Gebre and the Technical Director of the Carter Center's Trachoma Control Program is Dr. Paul Emerson. The focus of the country program has been on trachoma control, and delivery of quality eye care and blindness prevention programs to the majority of the population that live in rural areas (72%), largely un-served by trained eye care professionals.

The Carter Center is guided by a fundamental commitment to human rights and the alleviation of human suffering; it seeks to prevent and resolve conflicts, enhance freedom and democracy and improve health. The trachoma control program does not just fight disease, it fights the conditions that perpetuate disease: poverty, poor sanitation, lack of knowledge, and hopelessness. Together, the Carter Center and its program partners are working to build a brighter future for those at risk for this devastating disease**.**

**3.2 Duties & Responsibilities of Staff**

**3.2.1 Principal Investigator**

- Develop study design, specific aims and outcome measures, with help of biostatistician and study coordinators, partners.
- Obtain grant funding and with help of partners, develop grant budget
- Ensure that staff follow through on protocol and properly execute all areas of research
- Ensure that all ethical approval is maintained
- Write or add major contributions to all study-related publications
- Ensure proper masking procedure for staff involved in the study
- Supervise training certification for all trachoma examiners

**3.2.2 Study Coordinators (Proctor Foundation)**

- - - Ensure the execution of the study per protocol
    - Coordinate with the collaborating center, The Carter Center, Ethiopia (TCCE), particularly TCCE Study Coordinator in execution of the study
    - Manage correspondence between all collaborating organizations and parties
    - Maintain all ethical clearances for the study, including IRB renewals, ESTC and BUA and DSMC-related approvals
    - In collaboration with TCCE Study Coordinator, prepare all forms and documents necessary for fieldwork (randomized registration forms, exam sheets, fieldwork documents directing team, etc)
    - Train both Proctor and Ethiopian health worker team for each collection/treatment visit; direct team (with help of TCCE Study Coordinator) while in Ethiopia
    - Arrange logistics and itineraries for traveling team members in Ethiopia
    - Purchase, maintain, and organize transport of all necessary study supplies to/within Ethiopia
    - Coordinate and oversee shipping of collected study samples per protocol and IATA regulations
    - Maintain communication and partnership with Principal Investigator regarding all study activities and plans

**3.2.3 Study Coordinator (The Carter Center, Ethiopia)**

- - - At baseline, obtain permission from zonal health leaders to include each state team in the study. Meet with and obtain permission/consent from all kebele and sub-kebele leaders.
    - Supervise and train collection team throughout each collection, especially after departure of Proctor study coordinators
    - Train and supervise all Zithromax distribution teams
    - Train and supervise all census taking teams
    - Maintain all fieldwork documents and records
    - Complete all collection and treatment reports
    - Work with Data Coordinator to maintain treatment and census database
    - Oversee shipment of study samples after departure of Proctor team
    - Assist Proctor Coordinators in coordinating logistics in Ethiopia

**3.2.4 Co-Investigators (US & Ethiopia)**

- - - Assume responsibility for the study in the absence of the Principal Investigator
    - Supervise ophthalmic assistants, ophthalmic nurses, local health agents and other Proctor field team members in the field to ensure conformity to study procedures
    - Communicate with Study Coordinator and Principal Investigator to ensure the execution of the study as per the protocol

**3.2.5 Microbiologist**

- - - Train and supervise laboratory technicians for all lab procedures concerning the study, including Polymerase Chain Reaction (PCR) procedures
    - Perform all macrolide resistance testing
    - Follow standard laboratory procedures for all tests
    - Keep records of all tests performed
    - Maintain quality control measures as per approved standards
    - Make sure all equipment is calibrated and maintained
    - Maintain stock of laboratory reagents and supplies

**3.2.6 Data Coordinator (The Carter Center, Ethiopia)**

- With aid of TCCE Study Coordinator, create and maintain database for all ***treatment related data*** for the study
- Monitor correct receipt of treatment/census data from the ORBIS Study Coordinator using blue census books for each treatment or census visit
- Enter all data from treatment census books in clear database format for each visit and/or supervise data entry operators for any errors or omissions
- Analyze and provide data when requested by co-investigators or staff from Proctor Foundation
- Appropriately back-up all data
- Develop consistency checks in the data management

**3.2.7 Database Specialist (Proctor Foundation)**

- Create and maintain database for all ***collection and results-related data*** for the study
  - - Monitor correct receipt of exam sheets after each collection visit
    - Enter all data from exam sheets in clear database format for each visit
    - Supervise double entry to ensure accurate recording (first entry by Database Specialist, second entry by temporary Proctor staff)
    - Develop consistency checks in the data management—verify any inconsistencies or questions regarding exam forms through communication with Study Coordinator
    - Analyze and provide data regarding collection and results for study staff when needed, such as for publications or DSMC meeting
    - Back-up all data appropriately
    - Follow-up on any missing data or lab results
- Organize all study samples for PCR processing in the Proctor laboratory
  - - Monitor correct receipt of study samples (using corresponding exam sheets) after each monitoring visit
    - Prepare samples for PCR processing
    - Organize work flow of PCR processing for laboratory technicians, in consultation with Study Coordinator and Principle Investigator
    - Maintain all samples in storage freezers; create and maintain clear map of study samples for each freezer
    1. **Laboratory Technician (Proctor Foundation)**
- Pool and prepare all PCR samples, and process using COBAS Amplicor machine
- Perform regular maintenance checks of COBAS machine, under direction of microbiologist
- Report PCR results to Database Specialist
- Maintain cleanliness and maintenance of molecular biology laboratory and all equipment

**3.2.9 Biostatistician/Data Analyst at Proctor**

- Receive all data from the Data Coordinator/Study Coordinator TCCE and Database Specialist at Proctor Foundation.
- Review data for quality control purposes
- Ensure appropriate masking
- Prepare data analysis plan for annual Data & Safety Monitoring Committee (DSMC) meetings. Help analyze and prepare all presented data for DSMC meetings, DSMC reports, and all TANA study publications

**3.2.10 Treatment and Census Team: Local health extension workers from Ministry of Health, Ethiopia**

- During census phase, travel to enrolled state teams and obtain patient information as required for all Census forms
- During treatment phase:
  - - Complete training by TCCE Study Coordinator and officials from Local Zonal Health Desk regarding proper treatment protocols and procedures
    - Travel to enrolled state teams and administer study medication to the study subject per protocol
    - Counsel and motivate patients for follow-up and monitoring visits
    - Inform patients of available eye care facilities and procedures in local health centers and hospitals
    - Collect information on the nature of any Adverse Events experienced by study subjects, and report this back immediately to TCCE Investigators

**3.2.11 Collection Team: Local health workers/nurses from Ethiopian Ministry of Health**

- At each collection phase, complete on-going training and certification in clinical examination and collection procedures
- Prepare all study-related materials before travel to study sites
- In each state team, mobilize and identify all randomly selected participants
- Explain study purpose and procedure and obtain verbal consent for enrollment from each participant or participant guardian
- Under supervision of TCCE Study Coordinator and/or trained Proctor staff, perform clinical exam for each patient and collect all participant study samples according to protocol. Store and record all samples correctly for transport, organization, and processing
- Counsel and motivate patients for follow-up and monitoring visits
- Inform patients of available eye care facilities and procedures in local health centers and hospitals
- Collect information on the nature of any Adverse Events experienced by study subjects, and immediately report to TCCE Investigators

**3.3 Policy Matters**

**3.3.1 Protocol Revisions during the Trial**

Any changes to the protocol made during the course of the study will be incorporated in the revised protocol and the Manual of Operations and Procedures (MOP); any new forms will be incorporated in addendum. The protocol changes should be submitted and approved by the IRB of both the collaborating centers and by the Data and Safety Monitoring Committee (DSMC).

**3.4 Presentations and Publications**

All presentations and publications should include acknowledgement of the funding sources and give credit to the collaborating organizations and/or individuals involved.

**3.4.1 Authorship Policy**

**Papers Presenting Primary Results**

Acknowledgements will include grant source(s) and/or NIH grant(s), and the Data Safety and Monitoring Committee.

**3.5 Data Safety and Monitoring Committee**

A Data Safety and Monitoring Committee will be formed according to NIH guidelines. The Data Safety and Monitoring Committee will meet before the study commences and annually, and be observant for any severe and unexpected threats to the safety of the patients. They will have the authority to reduce any such threat, by discontinuing the enrollment of patients (were data to imply that these patients face unreasonable risk to antibiotics). As well as generally monitoring the safety and efficacy of treatment during the study, the DSMC will be empowered to enact formal stopping rules (see Stopping Rules, below).

**3.5.1 Stopping (and Restarting) Rules**

The WHO has recommended that trachoma programs revaluate their strategy after 3 years of implementation, but because we are monitoring intensely during this project, we have the opportunity to supervise treatment far more closely. In fact, we suspect from our previous studies that the treatments may be so effective that infection will be eliminated in some of the study communities long before 48 months. While there is an argument that continued mass treatments would prevent any reintroduced infection from taking hold, the balance between the risks and benefits of treatment will have changed. We will propose several types of stopping rules to the DSMC, all relatively conservative.

1) **Efficacy.** If an arm of Specific Aim 1 (biannual versus annual treatment) or Specific Aim 2 (treating children only versus treating everyone) is clearly superior in terms of both efficacy and resources, then the DSMC will have the opportunity to stop an arm. We will propose stopping rules to the DSMC at the pre-treatment meeting (using conservative, O’Brian-Fleming boundaries, with appropriate data and analysis provided by the biostatistician).

2) **Adverse effects.** Approximately 10 million doses of oral azithromycin have now been distributed for trachoma, and reports of serious side effects are essentially non-existent. This may be due in part to minimal surveillance. It also may be due to the fact these are extremely rare with a single dose of azithromycin. In fact, where carefully monitored, there were actually fewer Gastro-intestinal (GI) side effects after taking azithromycin.^127^ We will follow up on all reports of adverse events from local health centers/posts, as well as conduct a regular randomly selected survey to identify any possible post-treatment serious adverse effects (see Section 9.3). (Note that we are studying the resistance generated by antibiotic distributions in a separate grant, and all of this information will be made available to the DSMC). The DSMC will be given authority to discontinue treatments if the incidence of adverse events in treated arms becomes alarming.

3) **Stopping rule for individual state teams/communities.** If we are relatively certain that there is no ocular chlamydia left in a community, then continued treatment may not be warranted. If we are unable to identify ocular chlamydia in the sampled individuals of a state team on 2 consecutive visits, then the entire state team will be sampled on the subsequent visit. If no infection is identified, then treatment will be suspended. It’s important to note that monitoring will be continued, and treatment restarted as per the DSMC.

4) **Restarting treatment in Once-treated arm of Specific Aim 1.** One of the arms in Specific Aim 1 will be treated at baseline and then monitored. If the average prevalence of infection in children returns above 20%, which has been a WHO guideline for mass treatment, then antibiotics will be restarted in these state teams at the next scheduled visit. Note that previous studies have shown limited return of infection after even a single mass treatment, so we consider it ethical to monitor until it can be demonstrated that infection has returned.

**4. Patient Flow**

**4.1 Eligibility Requirements**

As part of The Carter Center/ITI trachoma control program, about 1,200,000 of the population in the Amhara area will receive azithromycin. For this study, those sub-kebeles (Government-defined groups of approximately 5 sentinel state teams) that are within 4 kilometers of the center of the town of Gandeweyn as determined by Global Positioning System (GPS) readings will be excluded. This is because state teams falling within this diameter are semi-urban areas which are thought to have a lower prevalence of trachoma (see Grant, page 49).

All age groups and sexes are eligible to receive azithromycin as per each study arm outlined above, except those contraindicated by Federal Ministry of Health, which currently are:

- Those self-reported as pregnant
- Children under six months old
- Those known to be allergic to azithromycin or macrolides such as erythromycin

These three exceptions will be treated with topical tetracycline eye ointment. Women uncertain of their pregnancy status may be offered an immediate-result pregnancy test if deemed appropriate by ethics committees.

**4.2 Randomization**

A list of 72 sub-kebele, randomly chosen from a list of 20 Kebele in the Amhara area, will be arranged for randomization. Twelve sub-kebele will be randomly assigned to each of the 6 study arms:

- Arm A: Annual treatment
- Arm B: Biannual treatment
- Arm C: Children-Only treatment
- Arm D: Delayed treatment
- Arm F: Annual treatment (without latrines)
- Arm G: Annual treatment (with latrines)

Three to five state teams exist within each of the 72 enrolled sub-kebele. One state team from each sub-kebele will be randomly selected to undergo monitoring at each of the study time points (by clinical examination and collection of samples for DNA amplification). In each state team and at each collection, sixty individuals from each of two age groups will be selected at random from the census for monitoring:

- 60 persons age 0 to ≤ 9. Referred to as “children” for study purposes.
- 60 persons age ≥10. Referred to as “adults” for study purposes.

The list of these 120 individuals will be randomly re-selected at each collection time point, as the study works to monitor prevalence at the community versus the individual level.

Please note that this is a change from the age groups in the Research Design Section of the Grant (1-5 year olds, etc., page 49 of Grant Text). Since Specific Aim 2 involves treatment of children aged 0 to ≤ 9 years old, monitoring of a random sample of individuals within and outside this age group would provide more valuable information for this study.

**4.3 Scheduling Visits**

The time schedule for examination and treatment is shown below in Table 2.

| **TANA Study** | | | | | | | | | | | | | | |
| --- | --- | --- | --- | --- | --- | --- | --- | --- | --- | --- | --- | --- | --- | --- |
| **Proctor Foundation/The Carter Center** | | | | | | | | | | | | | | |
| PCR Collection/Antibiotic Treatment Schedule: | | | | | | | | | | | | | | |
|  |  |  |  |  |  |  |  |  |  |  |  |  |  |  |
|  | **A** | **B** | **C** | **D** | **F** | **G** |  | **Minimum Total # Participants** |  |  |  | **Total RNA** | **Estimated PCR Collection Total** |  |
|  | **Annual** | **Bi-annual** | **Children Only** | **Delayed** | **Once** | **Once + Latrines** | **Total # of Arms** |  | **Total PCR Red** | **Total PCR Blue** | **Total NP** |  |  | **Date of Collection** |
| **TANA 1 Baseline** | Swab & Treat | Swab & Treat | Swab & Treat |  | Swab and Treat | Swab and Treat | **5** | 4,800 | 300 | 300 | 120 |  | **5,400** | June, 2006-completed |
| **TANA 2 Month 3** |  |  | Swab & Treat |  |  |  | **1** | 1,200 |  | 60 | 0 |  | **1,260** | October, 2006-completed |
| **TANA 3 Month 6** | Swab | Swab & Treat | Swab & Treat |  |  |  | **3** | 3,600 |  | 180 | 0 |  | **3,780** | Jan, 2006-completed |
| **TANA 4 Month 9** |  |  | Treat |  |  |  | **0** | 0 |  | 0 | 0 |  | **0** | NA |
| **TANA 5 Month 12** | Swab & Treat | Swab & Treat | Swab & Treat | Swab & Treat | Swab | Swab | **6** | 6,000 |  | 360 | 240 | **1,200** | **6,360** | May, 2007-completed |
| **TANA 6 Month 18** | Swab | Swab & Treat | Treat per Carter prgrm | Treat per Carter prgrm |  |  | **2** | 2,400 |  | 120 | 0 |  | **2,520** | Nov., 2007 |
| **TANA 7 Month 24** | Swab & Treat | Swab & Treat |  |  | Swab | Swab | **4** | 3,600 |  | 240 | 0 |  | **3,840** | May, 2008 |
| **TANA 8 Month 30** | Swab | Swab & Treat |  |  | Treat per Carter prgrm | Treat per Carter prgrm | **2** | 2,400 |  | 120 | 0 |  | **2,520** | Nov., 2008 |
| **TANA 9 Month 36** | Swab & Treat | Swab & Treat |  |  |  |  | **2** | 2,400 |  | 120 | 0 |  | **2,520** | May, 2009 |
| **TANA 10 Month 42** | Swab | Swab & Treat |  |  |  |  | **2** | 2,400 |  | 120 | 0 |  | **2,520** | Nov., 2009 |
| **Note: Swab only children in F and G, as primary outcome is prevalence in children** | | | | | | | **Total:** | **28,800** | **300** | **1,620** | **360** | **1,200** | **30,720** |  |

**Table 2. Treatment/Monitoring Schedule of Arms of Both Specific Aims**

**4.3.1 Baseline Visit**

At baseline, before treatment, all households in the sampled state teams were enumerated, and censuses of household members who are residents of the state team were recorded on the Census/Registration form (see Section 11.1). Monitoring during this visit was performed on a random sample of 60 people ages 0 to ≤ 9 years and 60 people age 10 and older. Monitoring consisted of clinical examination of the right upper tarsal conjunctiva using the WHO simplified trachoma grading (see Section 5.5), followed by swabbing of the conjuctiva. Samples were tested with DNA amplification for the presence of C. trachomatis infection. Upon completion of examination and swabbing, antibiotic treatment was distributed to the entire population.

**4.3.2 Treatment**

After the baseline census and monitoring/collection was completed, treatment was administered to all appropriate study state teams according to protocol. A treatment team, separate from the baseline enumeration and examination teams, adminstered the antibiotic. The treatment team consists of two local health extension workers (regional health staff) from each Kebele involved in the study. Each treatment cycle, this team participates in a training regarding antibiotic treatment and recording techniques. The training is taught by Carter Center staff and members of the Zonal Health Desk.

During the treatment phase, Health Extension Workers will seek out each person on the census, administer medication, and record whether or not each person has been treated. The treatment team will document the individual reasons for not being treated (e.g. death, temporary absence, permanent migration, refusal for treatment, etc.). The antibiotic dosages distributed will also be documented.

**4.3.3 Follow-up Visits**

Follow-up visits for all study arms are outlined in Table 2. At each monitoring visit, a random sample of 60 children age 0 to ≤ 9 years, and 60 adults, age 10 years and older in the appropriate study arms will be examined clinically. Swabs will be taken from these participants for DNA amplification in order to assess the presence of chlamydial infection. There will be no 2 month visit as mentioned in the Grant Text (this had previously been a time point that the WHO was interested in, although 3 months is now deemed acceptable for estimating efficacy of a mass treatment, because the return of infection has been demonstrated to be relatively slow).

State teams randomly assigned to the Delayed Treatment Arm will be

monitored only at the 12 month visit. After this collection, these state teams will exit the TANA study. The Carter Center will immediately treat these communities, and any further treatments will be part of the regular Carter Center treatment program in the Amhara region.

**4.4 Adherence to Treatment**

Adherence to azithromycin treatment will be essentially 100% of those treated since administration of the single dose of antibiotic is directly observed by The Carter Center treatment distribution team.

**4.5 Adverse Outcomes and Patient Death**

**4.5.1 Adverse Outcomes**

In the event of an adverse outcome, the patient will no longer be enrolled in the study and an alternative treatment for trachoma (e.g. tetracycline ointment) will be administered if the patient needs to continue treatment. Refer to Section 9.3 Adverse Reactions/Side Effects for detailed descriptions.

If a patient experiences a serious adverse outcome, they will be advised to alert the chairman of the state team, who will then inform the health care representative. This person will in turn inform The Carter Center site manager, who will contact the Co-Investigator in Addis Ababa. In addition, periodic reports on adverse events will be made to the Data Safety and Monitoring Committee.

All adverse events will be recorded on the census for each individual who experiences them, and they will be monitored as usual until the study is completed. If, for any reason, they will need further eye care, they will be referred to the nearest health center for examination and treatment, and the most appropriate action will be taken to provide immediate care.

**4.5.2 Patient Death**

The infant mortality rate is quite high in this area of Ethiopia. All deaths since the first census will be carefully recorded during at second census before the 12 month visit. Since the major causes of infant mortality in the area are diarrhea, respiratory infections, and malaria, it is possible that receiving two to four Azithromycin treatments per may actually have a positive effect. All death records (if available) will be maintained by the Data Coordinator at The Carter Center Ethiopia. The incidence of mortality for each study arm will be made available to the DSMC by the biostatistician.

**5. Examination and Procedures**

During registration and census-taking, each of the individuals from sampled state teams who are to be monitored for the study will be assigned a random identification number for laboratory and database anonymity. Examination of each individual will be performed as per the protocol outlined below:

**5.1 Registering Participants for Examination**

Prior to clinical examination the Registration Assistant will identify the participant, verify his/her age, and assign the next random identification number from the randomization list.

**5.2 Gloving of the Examiner's Hands**

Any hand that will touch a participant’s face or eyelids must be gloved for the examination. The examiner will put latex gloves on both of their hands prior to touching the participants’ eyelid, and a new pair of gloves will be used for each participant. Purell® Instant Hand Sanitizer will be available for hand sanitization.

**5.3 Examination Positions**

The examining position to be used in the field for young children will be the classic pediatric ophthalmic examination technique. With the aid of a helper seated directly opposite to the examiner, the child will be positioned with his/her head between the examiner's knees, with the child's face looking upwards toward the examiner. The legs of the child will be straddled across the helper and the arms held gently across the child’s chest. Care should be taken to keep the child’s eyes above the level of the examiner’s knees, in order to properly take the conjunctival swab.

For examining adults and older children, the participant should stand or sit facing the seated examiner, such that the participant's eyes are at the examiner's eye-level.

**5.4 Everting the Upper Eyelid**

For all participants, only the right upper eyelid will be examined for this study. The only exception to this is if the right eye is difficult to examine due to eye disease or injury, in which case the left eye will be examined.

In order to avoid passing contamination from the child into the eye, once the examiner dons a new pair of gloves, their gloved hands should not be used to position the child. The examiner uses their fingertips to grasp the central portion of the participant’s upper lid eyelashes. The upper lid is then everted, using a finger of the examiner's other hand (or the end of a sterile swab) as a fulcrum, positioned superior to the tarsal plate. The everted lid is held in place by the examiner's non-dominant hand holding the eyelashes against the orbital rim, thus keeping the examiner's dominant hand free for swabbing the participant’s tarsal conjunctiva later.

**5.5 Examining the Conjunctiva & Trachoma Grading**

The clinical examination will be performed by trained ophthalmic nurses/technicians, public health specialists or ophthalmologists. (See section 6.3 for information on grading and collection training). The examiner uses the 2.5X magnifying ocular loupes (Wilson Ophthalmics) to assess the tarsal conjunctiva of the everted upper right eyelid. The examiner will grade the conjunctiva according to the World Health Organization Simplified Trachoma Grading Scale, as shown in Table 3 below. If necessary, a hand-held torch light will be used by the examiner for illumination of the conjunctiva. The examiner tells the Trachoma Grade to the tuber, who then records the grade onto the Examination form.

Inter- and intra-examiner reliability will be performed, but not as a part of this study, as the clinical examination is not being used as an outcome.

***Table 3: WHO Simplified Trachoma Grading Scale***

**Definitions of the WHO Simplified Trachoma Grading Scale**:

**TF** (Trachomatous Inflammation – Follicular): the presence of five or more follicles in the upper tarsal conjunctiva.

**TI** (Trachomatous Inflammation – Intense): pronounced inflammatory thickening of the upper tarsal conjunctiva that obscures more than half of the normal deep tarsal vessels.

**TS** (Trachomatous Scarring): the presence of scarring in the tarsal conjunctiva.

**TT** (Trachomatous Trichiasis): at least one eyelash rubs on the eyeball.

**CO** (Corneal Opacity): easily visible corneal opacity over the pupil.

## Clinically active trachoma: defined as either TF or TI. If WHO guidelines recommend that TF alone is the most appropriate sign to follow, then we will easily be able to report this.

## Chlamydial infection (infectious trachoma): defined as positive PCR test for C. trachomatis.

**5.5.1 WHO Simplified Trachoma Grading Card**

The WHO simplified trachoma grading card will be used as the reference standard for grading trachoma in the field. The card shows color photograph standards of each trachoma grade, clear descriptions of each grading category, and the standard abbreviations for the each trachoma grade.

**5.6 Field Specimen Collection Quality Control Measures**

The diagram shows the stations for examination and swabbing of patients, designed for the most efficient patient flow and to minimize errors or confusion between team members. This diagram shows the Station Configuration for a four-person team.

Quality assurance for conjunctival swab collection will include taking duplicate swabs on a randomly chosen 5% of individuals.

**5.7 Examination Station and Diagram**

A diagram for examining and swabbing patients has been designed for efficient examination procedures and to minimize and errors or confusion between team members. This is depicted by the Station Configuration. It shows the station set-up of a four-person team.

**5.7 Examination Station Configuration**

**Examiner**

**Exam Seat**

(for adult Part. or village member assigned to

hold child Part.)

**Registration Worker**

Disinfectant

Child

Swabs/Gloves

**Tuber**

Gloves

Cooler Bag

Clipboard for Field Data

Trash Bag

**Figure 1**

**Examiner Assistant** (NP swabs only)

**6. Microbiology Laboratory Procedures for PCR**

**6.1 Specimen Collection for Microbiological Tests (Principal)**

Samples will be collected with reference to age, gender, household, and state team, but participant names will not be included in laboratory records. Samples will thus not be associated with the individual’s name, but with a 6-digit random identification number, masking laboratory personnel and preventing identification of the individuals infected. Lab results will not be available for weeks if not months, typically after the average duration of an ocular chlamydial infection. Thus all individuals will be treated according to their study arm, whether or not their lab tests reveal evidence of chlamydial infection.

**6.2 Materials for PCR**

**6.2.1 Swabs**

Specimens will be collected using sterile, individually-wrapped Dacron polyester-tipped swabs with a plastic shaft (manufactured by Fisherbrand®), as recommended by the COBAS AMPLICOR™ CT/NG protocol.

**6.2.2 Sample Tubes**

All field samples for DNA testing will be collected into sterile 2.0ml microcentrifuge tubes, manufactured by RPI®.

**6.2.3 Cooler Bags with Frozen Ice Packs**

Insulated cooler bags (California Innovations™) will be used to carry samples to and from the field. In addition, frozen gel ice packs designed to thaw slowly will be used to maintain the temperature in the cooler bags during transport.

**6.2.4 -20°C Freezer**

A standard -20°C freezer located at the Mota Health Center will be used strictly for the storage and freezing of ice packs and samples. This freezer is kept in a locked room on the grounds of the Health Center, which is under 24-hour security guard supervision.

**6.2.5 Latex Gloves**

Fisherbrand® latex examination gloves will be used to perform conjunctival examinations for trachoma. Each glove will be used once and never shared between participants. Used gloves will be collected in a trash bag and incinerated at the local Health Center incineration facility.

- 1. **Training of Examiners**
     1. **Pre-Monitoring Visit Training**

All specimen collectors and graders on the Proctor team new to the study will complete a three-hour training at the Proctor Foundation before departing for Ethiopia. The training will focus on 1) study background and rationale, 2) how to perform the clinical exam and 3) how to perform all collection procedures and protocols in order to standardize procedures among graders and tubers. The training will be led by the Proctor Study Coordinators, with input from the Database Manager and laboratory technician on proper collection and data recording techniques. The Principal Investigator will offer in-depth training on performing the clinical exam.

- - 1. **In-Country Training**

Before the start of the study, all local health workers assigned to complete study fieldwork underwent a series of rigorous trainings and certification in clinical grading and swab collection. At the start of each successive collection phase, examination team members (both local health workers from the Ministry of Health and Proctor staff) will participate in a full day of training at a staging site in the town of Gandeweyn, Ethiopia. The Principal Investigator and Study Coordinators will offer on-going training and supervision to all specimen collectors and graders in the field throughout the monitoring visit.

**6.4 Specimen Collection**

**6.4.1 Protocol for swabbing of the conjunctiva**

1. Upon taking a swab from the box, the tuber should announce what type of swab it

is:

- An unmarked “plain swab”, indicating no control or second swab collection necessary for this patient.
- A "blue air swab" (negative field control), indicating examiner must collect a second swab for this patient. Swab sachet will be marked with blue color.

The swab sachet should be opened in a sterile manner, revealing only the tip of the swab shaft, with the swab head itself remaining sterile deep within the sachet.

2. When ready to swab, the examiner should slowly pull out the swab (if removed too quickly the Dacron swab tip can unravel). The gloved hand should be held no closer than 1 inch from the Dacron polyester swab tip during the entire swabbing procedure, in order to avoid contamination of the swab tip.

3. As the examiner holds the right eyelid in the everted position, they swab the upper tarsal conjunctiva of the child with a gloved hand, using a steady and firm swab. Ideally the swab should be placed flat, with its entire length parallel to the conjunctiva to give the greatest surface area of contact, and if necessary, this may require repositioning of the child’s head by the examiner. The swab should be drawn firmly in one direction over the conjunctiva with enough pressure to cause blanching of the conjunctival vessels. The swab should then be rotated 120 degrees along its axis and the newly revealed fresh region of Dacron on the swab tip should now be drawn firmly across the conjunctiva. The swab should be rotated another 120 degrees along its axis and the conjunctiva swabbed for a third time. This will ensure sufficient collection of conjunctival epithelial specimen for PCR analysis in the lab.

**Note:** An aggressive twisting motion toward the cornea can push the conjunctiva upwards (towards eyebrow) which can lead to the swab falling onto cornea. Thus a pure lateral motion or slight twisting toward the eyebrow is preferable, in order to ensure safety of the cornea at all times. Quick movements should be avoided (less control, increased contamination, risk of touching the cornea). Instead, a slow, steady motion should be carried out. Care should be taken to avoid swabbing the eyelashes. Traction on the lower lid by the non-swabbing hand (or by an assistant) can keep the lower lid lashes from inadvertently touching the swab. Possibilities: The non-dominant (non-swabbing) hand should be placed on the patient’s forehead to give greater steadiness. This may also serve to alert the examiner to any movement by the patient (tactile instead of just visual). Also, this action will help steady the head of a child in case of movement.

**6.4.2 PCR Control Swabs**

A negative field control ("blue air swab") will be taken on a randomly chosen 5% of monitored children to assess the frequency of contamination. For each negative field control, the examiner will pass a sterile Dacron swab within 1 inch of the child’s conjunctiva. Control swabs are taken after the original swab but before changing gloves for the next patient.

Both negative field control swabs and duplicate control swabs ("red double swabs") were taken during the baseline visit. (Duplicate controls are used in order to validate the PCR test). During the 2006 TANA Data Safety and Monitoring Committee Meeting, the committee agreed that duplicate field controls were no longer necessary, after researchers showed consistent 95% concordance between duplicate field control swabs in PCR testing. Only negative field controls will be taken for all post-baseline TANA visits.

**6.4.3 Protocol for tubing and handling of samples**

The tubing and handling protocol must be carefully followed in order to prevent contamination and ensure the safe transport of the samples back to the microbiology laboratory from Ethiopia to the Proctor Foundation. The person in charge of labeling, tubing, arranging, and handling the samples needs to perform this task in the most orderly and attentive manner.

1. Both hands of the tuber should be gloved at all times. The tuber's gloves only need to be changed when any potential contamination of the gloves occurs. The tuber opens the capped, hinged lid of a microcentrifuge tube, which has been labeled with the participant’s random identification number.

2. The swab is inserted by the examiner (using the still-gloved hand that swabbed the participant’s conjunctiva) into the microcentrifuge tube held by the tuber. The swab shaft should only be inserted until the Dacron swab head is fully in the tube. The tuber should lower the cap onto the swab shaft held by the examiner, and the examiner should quickly break the swab shaft using a swift downward snapping wrist movement (this will be demonstrated in the field the first day).

3. The tuber should screw the cap of the microcentrifuge tube tightly and place it in the sample collection box, located in the cooler bag filled with frozen ice packs. The flap of the cooler bag should be closed between each patient. The cooler bag should be in as cool a place as possible in the field, in a shaded area out of the sun.

4. Upon returning from the field each day, the samples will be immediately taken to the Mota Health Center and stored in a commercial -20°C freezer, reserved solely for storage of specimens and ice packs. All samples will be in sample boxes, labeled with the state team and district names for easy future identification.

**6.5 Shipping and Storage of Samples**

In accordance with the Roche COBAS AMPLICOR™ CT/NG protocol, swab samples taken in the field will be transported on ice in a closed, insulated container until arrival in San Francisco, where they will be stored at -80°C for later analysis. Samples will be imported in the USA as per the CDC Permit to Import/Transfer Etiological Agents or Vectors of Human Disease.

- - 1. **Materials for shipping**
- Insulated shipper box (ThermoSafe VIP insulated shipper, 615DCS or 499 DCS)
- Gel Packs (ThermoSafe U-tek 24 oz. -10°F)
- Fisherbrand Exact-Temp temperature dataloggers
- Shipper label
- Consignee label
- Responsible person label
- UN3373 label
- Biological substance, Category B label
- Air Sea BioBag-2 95kPa specimen bags (only if you have samples containing liquid)
- Documentation to include in shipper box
  - Pro-forma invoice (prepared by Proctor and completed by Carter Center)
  - Letter from Carter Center (signed by Ato Teshome)
  - CDC permit
  - Letter of approval from ESTA
  - DHL Airway bill (obtained and prepared at DHL)

**6.5.2 Packing the samples**

For samples containing liquid media, such as NP swabs:

- Absorbent material (clean paper towel or cotton pad) should be placed inside the sample box to check for any media leakage.
- Boxes should be securely wrapped with clear plastic wrap and then clear plastic tape.
- Place all sample boxes with media in pressure-resistant bags (Air Sea BioBag-2) and seal according to written instructions on each bag. BioBag-2 can take up to two 100-tube sample boxes.
- Sample boxes may also be wrapped with a cotton absorbent pad (chuck) for improved insulation.

For dry samples without media:

- Boxes can be wrapped with plastic wrap and clear plastic tape only. There is no need to use pressure-resistant bags.
- Boxes may also be wrapped with a cotton absorbent pad (chuck) for improved insulation.

For all sample types:

- As soon as samples are prepared for shipping, they should be returned to the -20°C freezer to prevent samples from thawing.
- Place all stickers on outside of the shipper box (see detailed shipper instructions).
- Leave at least one shipping box, preferably a partially-filled one, unpacked for inspection by the DHL employee.

When ready to ship, the prepared sample boxes and the datalogger should be placed into the partially filled shipper box. Set the datalogger device with delay-start option and 30-minute reading interval, and place in a small ZipLoc bag to protect from condensation.

Next, all frozen icepacks and samples should be placed in the shipper box. (6 gel packs per a small shipper, 12 gel packs per a large shipper)

- - - For a small shipper box: Use 6 frozen gel packs-- one below sample boxes, 4 on each side, and one on top of sample boxes
    - For a large shipper box: Use 12 frozen gel packs-- 6 gel packs flat at the bottom and 6 on the top

Immediately after the shipment is accepted by the DHL representative, the shipper box should be closed and taped shut using clear plastic tape. Tape should be placed around all corners of the outside box.

**6.6 Methods**

Laboratory testing is the current standard of care for the identification of *C.trachomatis* infections in the U.S. After collection, all samples shipped from Ethiopia will be filled with 1 ml of M4RT media at the Proctor laboratory, and tested for *C. trachomatis*.

**6.6.1 Description of the Roche COBAS AMPLICOR™ CT/NG Assay**

The conjunctival swabs will be tested for the presence of *C. trachomatis* using the Roche COBAS AMPLICOR™ CT/NG assay. This assay is a qualitative *in vitro* test for the detection of *C. trachomatis* plasmid DNA. The assay is based on four major processes; specimen preparation: PCR amplification of target DNA using CT specific complementary primers; hybridization of the amplified DNA to oligonucleotide probes specific to the target; and detection of the probe-bound amplified DNA by colorimetric determination.

**6.6.2 Processing of Samples for PCR**

Samples are handled as per COBAS AMPLICOR™ CT/NG sample processing protocol, with the following modifications:

1. Samples are boiled for 10 minutes at 100°C. Boiling of samples is an accepted treatment method to remove substances that may be inhibitory to the PCR amplification process.
2. Samples are pooled.

**6.6.3 Procedure for Masking Samples**

In order to mask the location origin, the control status, and the clinical exam grading of the conjunctival samples collected in the field, the Database Manager at Proctor will assign an identification number different from the random number assigned to each child. The PCR results will be recorded according to this laboratory identification number, thus masking the lab until all the samples have been processed. The Database Manager will then link the lab ID number to the random sample number to reveal the test results by state team.

**6.6.4 Procedure for Pooling Samples**

At baseline, all samples will be processed individually. At post-treatment visits, samples will be divided by state team and age group (0 to ≤ 9 years and 10 and older). Within a state team and an age group, samples will be randomly pooled 5 per tube as described below (note that if the number of samples in a group is not divisible by 5, then a single 4-pool, 3-pool, 2-pool, or unpooled tube may be necessary). If, in a state team, the proportion of pooled positives is greater than 50%, then samples will be re-pooled with 2 per tube, and the PCR repeated. Analysis of pooled results is described in Section 2, “Pooling of chlamydial tests”.

In order to pool the conjunctival samples in the lab, the microbiology lab staff at Proctor will assign a new pool ID number for every sample, and samples will be stored at -80°C freezer until PCR testing (if not processed that day).

**6.7 Quality Control**

1) A *C. trachomatis*(+) control and a *C. trachomatis*(-) control is included in each test run of the COBAS AMPLICOR™ CT/NG.

2) To test the effect of sample processing, a known positive sample is processed and tested in each test run. (This control is helpful when testing large numbers of negative samples.)

3) An internal control intended to identify specimens that contain polymerase inhibitor is run routinely on each sample. The internal control helps identify false negative results.

**6.8 Laboratory Results Reporting**

All lab results will be kept in computer files as well as in hard-copy form by the Database Manager. The principal investigator and the DSMC will be updated regularly on the progress of the lab work throughout the course of the study.

**7. Macrolide Resistance Testing**

**7.1 Specimen Collection for Macrolide Resistance Testing**

Nasopharyngeal samples will be collected with reference to age, gender, household, and state team, but participant names will not be included in laboratory records. Samples will thus not be associated with the individual’s name, but with a 6-digit random identification number, masking laboratory personnel and preventing identification of individuals.

**7.2 Materials for NP Collection**

**7.2.1 Swabs**

Specimens will be collected using sterile, individually-wrapped pediatric calcium alginate swabs with a malleable metal swab shaft for patient comfort and safety (manufactured by Fisherbrand®).

**7.2.2 Sample Tubes**

All field samples for DNA testing will be collected into sterile 2.0ml microcentrifuge tubes, manufactured by RPI®.

**7.2.3 Cooler Bags with Frozen Ice Packs**

Insulated cooler bags (California Innovations™) will be used to carry samples to and from the field. In addition, frozen gel ice packs designed to thaw slowly will be used to maintain the temperature in the cooler bags during transport.

**7.2.4 -20°C Freezer**

A standard -20°C freezer located at the Mota Health Center will be used strictly for the storage and freezing of ice packs and samples. This freezer is kept in a locked room on the grounds of the Health Center, which is under 24-hour security guard supervision.

**7.2.5 Latex Gloves**

Fisherbrand® latex examination gloves will be used to perform nasopharyngeal examinations. Each glove will be used once and never shared between participants. Used gloves will be collected in a trash bag and incinerated at the local Health Center incineration facility.

- 1. **Training of Examiners**

The first day of fieldwork is a training day for all specimen collectors and graders on the Proctor and Carter Center Ethiopia teams. (See section 6.3.2). The Principal Investigator and Study Coordinators will offer on-going training and supervision to all specimen collectors and graders throughout the monitoring visit.

- 1. **Nasopharyngeal Specimen Collection**
     1. **Protocol for Nasopharyngeal Sample Collection**

During collection visits that include nasopharyngeal sample collection, 15 swabs in the box will be marked to indicate participant selection for NP sample collection.

1. In this case, upon taking a DNA swab from the box, the tuber should announce

what type of swab it is:

- An unmarked “plain swab”, indicating no control or second swab collection necessary for this patient.
- A "blue air swab" (negative field control), indicating examiner must collect a second swab for this patient. Swab sachet will be marked with blue color.
- (If included) A "red double swab" (duplicate control), indicating examiner must collect a second swab for this patient. Swab sachet will be marked red.
- An “NP swab”, indicating examiner must also collect a nasopharyngeal swab for this patient. Swab sachet will be marked green.

1. If the swab is an “NP swab” marked green, a trained team member will

immediately be called to assist the exam team. Standing behind the examiner, the assistant will select a pediatric calcium alginate swab (manufactured by Fisherbrand®), and open the swab sachet in a sterile manner (revealing only the tip of the swab shaft, with the swab head itself remaining sterile deep within the sachet). Immediately after completion of the conjunctival eye swab collection (and if necessary, control swab collection), the examiner will remove the swab from the sachet and place the tip down the participant’s nasopharynx.

1. The examiner will quickly rotate the swab 120° three times back and forth, and then remove the swab from the nose.
2. The examiner will place the swab in a tube containing 1.0 mL of STGG (skim milk, tryptone, glucose, and glycerin) media. The assistant will cut the handle off using sterile scissors (cleaned with alcohol pads between participants). The tuber will close the cap of the STGG tube with the swab immersed.

**7.5 Transport and Storage of Samples**

The nasopharyngeal swab samples in STGG will be initially stored in the field at 4°C using an insulated storage bag filled with Fisher brand ice gel packs and then transferred to -20°C.

All Nasopharyngeal samples will be transported on ice in a closed, insulated container via DHL until arrival in San Francisco, where they will be stored at -80°C for later analysis. Samples will be imported in the USA as per the CDC Permit to Import/Transfer Etiological Agents or Vectors of Human Disease. (See section 6.5)

**7.6 Methods**

The nasopharyngeal swab for pneumococcal resistance testing will be transported and processed using standard microbiological techniques at Proctor Foundation Microbiology lab.  Isolates will be confirmed *S. pneumoniae* by growth on *Streptococcus*-selective media, alpha-hemolysis, susceptibility to optochin and bile salt solubility.  MIC values for azithromycin and trimethoprim/sulfamethoxazole will be determined using standardized broth dilution.

**7.6.1 Procedure for Masking Samples**

The microbiologist and all lab staff will be masked to the state-team of origin.

**7.6.2 Quality Control**

Control isolates will be used to confirm the reliability of antibiotic susceptibility testing.

**7.6.3 Laboratory Results Reporting**

All lab results will be kept in computer files as well as in hard-copy form by the Microbiologist.

**8. Clinical Photography**

**8.1 Photography Protocol**

To measure accuracy of grading and inter-grader reliability, photographs will be taken of the upper right tarsal conjunctiva of a subset of children examined for conjunctival swabbing. Clinical photography will be performed before conjunctival swabbing. A handheld Nikon D70 digital camera with a macro lens (1:1) will be used. This causes no damage to the eye, is well tolerated by children, and is a standard clinical procedure at UCSF.

**8.2 Grading of Clinical Photographs**

A trained grader at the Proctor Foundation, UCSF, will grade the photograph of the conjunctiva according to the WHO simplified trachoma grading scale as either active (TF or TI) or inactive. The grader will be masked as to the treatment given.

**9. Study Medication**

**9.1 Study Medication Description (from Pfizer, Inc.)**

Zithromax® is supplied for oral administration as film-coated, modified capsular shaped tablets containing azithromycin dehydrate equivalent to either 250mg or 500mg azithromycin and the following inactive ingredients: dibasic calcium phosphate anhydrous, pregelatinized starch, sodium croscarmellose, magnesium stearate, sodium lauryl sulfate, hydroxypropyl methylcellulose, lactose, titanium dioxide, triacetin and D&C red #30 aluminum lake.

Zithromax® for oral suspension is supplied in bottles containing azithromycin dehydrate powder equivalent to 300mg, 600mg, 900mg, or 1200mg azithromycin per bottle and the following inactive ingredients: sucrose; sodium phosphate, tribasic, anhydrous; hydroxypropyl cellulose; xanthan gum; FD&C Red #40; and spray dried artificial cherry, crème de vanilla and banana flavors. After constitution, each 5mL of suspension contains 100mg or 200mg of azithromycin.

**9.2 Dosage Information**

Azithromycin will be administered as a single dose, in tablet form for adults and in oral suspension form for children. Dosing will be as per the WHO recommendations for treatment of active trachoma:

- Single dose of one gram of azithromycin for adults
- Single dose of 20mg/kg in children (up to the maximum adult dose of 1g)
- Height-based dosing of children will be acceptable, as per The Carter Center’s program—note that this is supported by the WHO PBD group.

Individuals who are either under the age of 6 months, pregnant, or allergic to macrolides/azalides will be treated with 1% tetracycline eye ointment to be applied twice daily to both eyes for a 6 week period. If the appropriate ethical committee in Ethiopia suggests pregnancy tests for self-reported pregnant women, or if the women are unsure of their pregnancy status, then they may be offered an on-site pregnancy test.

**9.3 Adverse Reactions/Side Effects**

Approximately 10 million doses of oral azithromycin have now been distributed for

trachoma, and reports of serious side effects are essentially non-existent. This may be due

in part to minimal surveillance. It also may be due to the fact these are extremely rare with

a single dose of azithromycin. In fact, where carefully monitored, there were actually fewer

GI side effects after taking azithromycin. We will create a network to identify any possible

post-treatment serious adverse effects.

Azithromycin is generally well-tolerated. The most common side effects of azithromycin and erythromycin are diarrhea or loose stools, nausea, abdominal pain, and vomiting, each of which may occur in fewer than one in twenty persons who receive azithromycin. Rarer side effects include abnormal liver function tests, allergic reactions, and nervousness. Diarrhea due to *Clostridium difficile* has been rarely reported.

The **adverse reactions** that may occur after taking azithromycin will be explained to individuals prior to enrollment in this study. All individuals who have been given azithromycin will be told to immediately communicate side-effects to representatives of The Carter Center program, which will ensure that appropriate medical care will be provided, and that the frequency and severity of adverse events can be assessed. Additionally, adverse reactions to azithromycin will be monitored by local health workers through an adverse event questionnaire. The questionnaire is to be administered at least 24 hours after medication is received, as side effects may not otherwise be evident.

All health workers will be trained in survey administration methods, including:

1. Getting informed consent
2. Accurately selecting from the randomly selected list households to interview
   1. Working from a list of 14 households in each state team, health workers will be encouraged to interview the first 10 households on the list, only moving through the list if someone is not home or is unavailable
   2. Health workers will be advised to go through the list in order rather than simply choosing the easiest households to reach
3. Remaining neutral when asking questions (i.e. asking the question exactly as it is written on the paper in a neutral tone of voice, as not to lead the respondent or suggest bias)

120+ households will be randomly selected to participate in the questionnaire.

- 12 sentinel state teams selected from the 48 sentinel state teams in Arms A, B, C and D
- 10 households selected from each sentinel state team

**9.4 Treatment Costs**

The azithromycin (Zithromax®) has been donated by the Pfizer Corporation free of charge. There will be no costs to acquiring the study medication.

**9.5 Alternate Therapies (Control Villages)**

Control arms will be administered azithromycin immediately after they are swabbed.

**9.5.1 Tetracycline Ophthalmic Ointment**

Tetracycline ophthalmic ointment (1%) is the current standard treatment in Ethiopia for ocular trachoma, and will be distributed to study patients who are not eligible to receive azithromycin.

**9.6 Treatment/Monitoring Schedule**

The treatment and monitoring schedule for all study arms is shown in Table 2 (see Section 4).

**9.7 Medication Procurement/Donation**

Pfizer, Inc. will provide the donation of Zithromax® (azithromycin), which will be shipped directly to Ethiopia and received by a representative of the Ethiopian Ministry of Health, who will manage the customs process and transport the medication from the port to a storage site. The exemption of duties and taxes will be settled by the Ethiopian Customs Authorities and the Ethiopian Ministry of Health.

**9.8 Study Medication Storage and Accountability**

Zithromax® tablets will be stored between 15° to 30°C (59° to 86°F), as recommended by the Pfizer Labs. A record of the exact number of tablets distributed and quantity of oral suspension dispensed will be kept by The Carter Center treatment distribution team.

**9.9 Medication Quality Control**

Study medication will be stored in The Carter Center project office prior to use. The ophthalmic assistant and the study coordinator will regularly check and record the study medication expiry dates. The expiration dates on the medication containers will be strictly monitored and all expired study medicine will be discarded appropriately.

**9.10 Checking Antibiotic Coverage**

In order to observe the antibiotic coverage of participating state teams, the ophthalmic nurses and public health nurses who participate in the antibiotic distribution will mark the dorsal aspect of the right hand of all treated individuals in a randomly chosen number of state teams. The treatment distribution team will then return to the treated state team the following afternoon or the next day, to survey every household to determine the number of marked individuals in each state team. This number is then compared to the total population in each state team from the census to establish an antibiotic coverage level. The return visit to the treated state teams provides an opportunity to identify and treat any individuals who missed the antibiotic distribution initially.

**10. Protection of Human Subjects**

**10.1 Internal Review Board Approval**

As part of the trachoma elimination program, the registration process of azithromycin by the Drug Administration and Control Authority, Ethiopia, has already been finished. The mass distribution of the azithromycin plan is prepared together with the Zonal and Regional Health authorities.

**10.1.1 UCSF Committee on Human Research**

The University of California, San Francisco, Committee on Human Research will annually review study protocol for ethical approval.

**10.1.2 Ethiopian Science and Technology Commission**

The study protocol has been reviewed and granted ethical approval by the Science and Technology Commission of Ethiopia.

**10.2 Informed Consent**

The chairman of each state team has given permission to include the state team in the study. Additionally, the study was initially discussed with all adult family members in the state teams by the participating Carter Center staff members who speak Ethiopian Amharic or the local language.

At each collection visit, all adults 16 years of age and older will be informed about the possible risks and benefits of the treatment and swab taking, and asked to give a written consent. Young adults and children below 16 years of age, who cannot give consent by law, will be included in the study only following the receipt of verbal informed consent from a parent or guardian. If, at any time, a parent or guardian elects to withdraw themselves or a family member from the study, it will be made clear that they will be offered the same medical treatment outside the study.

**10.3 Adequacy of Protection Against Risk**

There are several layers of procedures to help minimize study-associated risk to participants. The risk of antibiotic treatment will be minimized by treating only those who fit in the approved age and inclusion category, as well as by regularly scheduled follow-up examinations by a trained trachoma grader. Should the antibiotic be ineffective to an individual, the study medication will be discontinued for them. In the event of any adverse effects, appropriate medical care will be provided by the local health center.

**10.4 Inclusion of Pregnant Women & Children**

All participants, regardless of gender, will be accepted. Pregnant women will be excluded from receiving oral azithromycin, and will be offered topical tetracycline eye ointment in its place.

All participants will be included in the study. Appropriate verbal assent will be obtained from all participants over the age of 10 entering the study. Appropriate verbal consent will be obtained from parent/guardians of children <18 years of age entering the study. (See Appendix 13.1)

**10.5 Compensation to Participants**

There is no cost to the participant and there is no reimbursement for overall participation in this study. Each participant will receive free ophthalmic examinations during the course of the study.

**11. Data Collection & Management**

Approximately 100,000 of the population of 250,000 in the Amhara area will receive azithromycin. A census will be conducted before distribution of the drug to avoid people coming from the adjacent areas for treatment and to gauge the coverage of antibiotic treatment at each distribution. Seventy-two sub-kebele will be randomized between the 6 study arms for treatment: Annual, Biannual, Children-only treatment, Delayed treatment, Once treated (without latrines) and Once treated with latrines. We will treat each sub-kebele (3-5 state teams) as a unit, although we only monitor one randomly selected state team from each sub-kebele.

In order to standardize procedures among the examiners, training will be given in advance on the grading system and the swab collection protocol (See section 6.3.2). The Carter Center Study Coordinator, with help from the Proctor Study Coordinators, will be responsible for the overall day-to-day supervision of the census, collection and treatment teams in the field, as well as the completion of all forms and proper recording, storage and transport of study samples.

The baseline visit began with the enumeration/census taking of each enrolled state team, followed by examination and swab collection. In order to ensure time comparability, the team will work to complete each collection phase in 2-6 weeks. After the examination/collection phase, the distribution of azithromycin (according to study protocol) will be completed in 2-3 weeks time. Swab collections are to be performed +/- 1 month from the target date.

All census and treatment data will be entered in Ethiopia by the TCCE data entry team within one month of return from the field. (Coverage levels will be measured directly, relative to the census.) All data from the exam sheets (random number, clinical exam result, gender, age) will be entered at Proctor by the Database Specialist within one month of return from the field. All PCR results will be double-entered at Proctor within one week of obtaining results. Any inconsistencies will be resolved by the appropriate study coordinator (The Carter Center or Proctor), and if necessary, by consultation with the appropriate examiner or lab personnel. Patient names will not be made available to UCSF personnel, individuals will be identified by ID number only. All data will be backed up upon entry, and forms will be kept in locked cabinets through the conclusion of the study.

At baseline, quality assurance included duplicate swabs on a randomly chosen 5% of participants, and negative field control swabs taken on a different 5% of participants, as we have done in our preliminary studies. At the 2006 DSMC meeting, the committee advised the team to stop collecting duplicate swabs, as 1) They may have been confusing the collection team and causing higher rates of contamination in the negative field control swabs and 2) past studies and results from baseline both showed more than 95% concordance between duplicate swabs. For all visits after baseline, the team will collect 5% negative field controls only.

Pooling requires knowledge of which samples are controls and which are not. Organization and masking of samples for pooling is performed by the Database Manager only. Pooling and PCR testing is performed by the Laboratory Specialist only.

**11.1 Census Administration**

Basic protocols for census taking are available in the Amharic language.

**11.1.1 Form Description**

The census form (census book) that will be used in this study will be prepared by The Carter Center Ethiopia and printed in Amharic. Each page will represent one household and contain fields for the household number, date recorded and recorder’s name. Columns for each individual’s name, age and gender, and medication delivered at each treatment phase will be provided.

**11.1.2 Completing the Census Form**

The census form will be completed in Amharic by a census team selected by The Carter Center Ethiopia. The census team will visit each state team and inform the chairman to alert each head of household in the village. Each head of household will be interviewed to provide the full name, age, and gender of each member of the household. Each household will be assigned a number and each individual within a household will also be assigned a number.

**11.2 Field Data Collection**

**11.2.1 Registration Form**

A randomized Registration Form is created for each state team. The Registration Form lists the names of 120 children (age 0-5) randomly chosen from that State Team’s census, and includes each child’s age, gender and head of household. Names are chosen randomly using the Excel program and written entirely in Amharic. If the census lists less than 120 children age 0-5, all children’s names in that age group are chosen. Please note: 120 names are chosen to ensure that at least 100 children (where available) may be identified for swabbing.

A new randomized registration form is generated for each state team at each collection visit and is used for both mobilization (the day before collection is scheduled) and during registration the day of the collection.

**11.2.2 Examination Form**

The Examination Form contains columns for placing the random numbered stickers for each sample collected in the field, age, gender, and the clinical exam of each patient. State team information, date, GPS location and the names of examination team members are also included at the top of each form.

**11.3 Data Forms Management**

All completed Registration Forms, Examination Forms and Treatment/Census books will be given to The Carter Center Study Coordinator at the end of each workday and stored in a safe, secure place for transport to Addis Ababa. The Study Coordinator will check the forms for completeness and accuracy.

- **Treatment/Census Books:** Treatment coverage calculated and recorded, books organized and stored in secure place at TCCE office in Addis Ababa.
- **Exam forms:** Should be immediately photocopied, with one copy left stored with TCCE, and the other transported to the Proctor Foundation for double data entry under supervision of the Data Management Specialist.
- **Registration Forms:** Forms have patient information/names and will be organized by TCCE Study Coordinator and stay in Ethiopia at TCCE office, stored in secure, locked area.

**11.3.1 Data Editing**

Before data entry, the forms will be reviewed and cross-checked for consistency and completeness. If the forms are not filled out completely, the Study Coordinator (TCCE or Proctor) will contact the person responsible for completing the form to provide missing data or clarify any inconsistent data. The Study Coordinator is the only person who is authorized to add missing data or make any changes to the study forms. All changes will be made in a different color ink and initialed and dated.

**11.3.2 Data Entry and Quality Control**

The treatment data collected in the field will be entered into a database at The Carter Center Ethiopia office. A double entry will be done to minimize data entry errors. The study coordinator will do the final editing. The collection fieldwork data will be entered into a database by Proctor Foundation’s Database Manager, who will also oversee the work of the staff member helping to double-enter the data. The Proctor Database Manager will also enter all PCR results into the database.

**11.3.3 Data Entry Errors**

A data manager enters the forms and will then verify the forms and re-enter the data. The corrected data entry error file will then be electronically merged with the full database to produce the corrected file.

**11.3.4 Data Consistency & Validity**

Through range checks, the data entry software ensures to a large extent that there are no inconsistencies or invalid data. Data will also be checked by those entering and Database Manager for consistency and errors.

The software has been developed for checking the consistency. The software will create an error file with relevant data such as the form identification, field names and the data. The data manager will then consults the forms, resolve the inconsistency and enter the correct data. The corrected consistency error will then be electronically merged with the full database to give the corrected file.

**11.4 Data Security**

The entry and corrections will be made only through the program. It will not be necessary to do anything directly with the database. This will ensure that the data will not be mishandled at any point in time. Only the Database Manager and one designated data analyst can access the data in the main computer.

**11.5 Data Storage**

At the data-coordinating center, the operator takes a backup copy of the day’s entry onto the data server and a CD. The data in the main computer will be backed-up daily and there will be at least two sets of backup disks at any particular time. All the backup CDs will be kept in a place different from the computer center.

**11.6 Data Analysis**

**Estimation of disease prevalence.** PCR results will be tabulated by state team. For individual PCR tests (at baseline), the prevalence in 0 to ≤9 year olds and in those ≥ 10 is determined directly. For pooled results, we use maximum likelihood estimation to determine what prevalence in the population (by the 2 age-groups, by state team) has the highest likelihood of resulting in the observed pooled results (the likelihood is a relatively simple function of the number of positive and negative 5-pools, positive and negative 4-pools, etc., with appropriate combinatorics) Note that this is a modification from the grant text (Equation 1, page 37: this equation can only provide an estimate if all pools are made up of the same number of samples).

**11.7 Data Management, Security and Quality Assurance**

All treatment and collection data will be entered within four working weeks of collection. Data will be double entered and inconsistencies will be resolved by the respective study coordinator or, if necessary, by consultation with the appropriate data collector. Access to any patient information will be protected by a password, or locked in a secure storage room**.**  All data will be backed up upon entry, and forms will be kept until the conclusion of the study. The DSMC can make requests to have access to the data at any point during the course of the study.

**12. Statistical Analyses**

For analysis of arms A and B, a separate comparison at each of the three time points (18 months, 30 months, and 42 months) will be used. The prevalence of PCR infection will be analyzed with the pooled estimate for each village using ANCOVA, testing the null hypothesis (two-sided, alpha=0.05) that there is no difference in prevalence between arms A and B, while adjusting for the baseline prevalence. We will not be reducing the p-value at each time point. This is the same analysis we will use for the comparison in arms F and G (latrine usage, one time point.)

For secondary/exploratory analysis, a repeated-measures ANOVA (in which we will adjust for the baseline prevalence and treatment arm) will be used, assuming an AR(1) error structure.

**12.2 Inter-observer Variability**

Although inter-observer variability is a large concern with grading trachoma in the field, it should be noted that the clinical exam is NOT an outcome of this study. For other, programmatic purposes, inter-observer variability will be estimated by having all examiners grade a series of patients, masked to their colleagues’ grading. The clinical examination in the field will be made by trained and qualified trachoma graders (public health nurses, ophthalmic assistants, and ophthalmic nurses), who have undergone a formal training session (and, in most cases, a number of refresher sessions) and have been selected to work in the field based on their grading accuracy.

Lab tests will include a negative field control on 5% of individuals (blue control). Previously we have found these to be ~99.1% negative, indicating a small amount of field contamination or false positive PCR testing—note that the specificity of the Amplicor test is estimated to be ~99.5%.

**13. Appendix**

**(Please see attached documents)**

**13.1 Patient Verbal Consent Scripts**

**13.2 Randomized Registration Form**

**13.3 Examination Form**

**14. References**

1. Henderson DA. Eradication: lessons from the past. Bull World Health Organ 1998;76 Suppl 2(4):17-21.

2. Taylor HR, Pacqué M, Muñoz B, Greene BM. Impact of mass treatment of onchocerciasis with ivermectin on the transmission of infection. Science 1990;250(4977):116-8.

3. Farid M. The malaria programme--From euphoria to anarchy. World Health Forum 1980;1:8.

4. Dowdle WR. The principles of disease elimination and eradication. Bull World Health Organ 1998;76 Suppl 2(10):22-5.

5. Wawer MJ, Gray RH, Sewankambo NK, et al. A randomized, community trial of intensive sexually transmitted disease control for AIDS prevention, Rakai, Uganda. AIDS 1998;12(10):1211-25.

6. Wawer MJ, Sewankambo NK, Serwadda D, et al. Control of sexually transmitted diseases for AIDS prevention in Uganda: a randomised community trial. Rakai Project Study Group. Lancet 1999;353(9152):525-35.

7. WHO. Report of the First Meeting of the WHO Alliance for the Global Elimination of Trachoma. Geneva, Switzerland: World Health Organization, 1997.

8. Report of the First Meeting of the W.H.O. Alliance for the Global Elimination of Trachoma. Geneva: World Health Organization, 1997.

9. Lietman T, Porco T, Dawson C, Blower S. Global elimination of trachoma: how frequently should we administer mass chemotherapy? Nat Med 1999;5(5):572-6.

10. Thylefors B, Négrel AD, Pararajasegaram R, Dadzie KY. Global data on blindness. Bull World Health Organ 1995;73(1):115-21.

11. Lietman T, Dawson C, Osaki S. Ocular chlamydial infections. Int Ophthalmol Clin 1998;38(4):125-35.

12. Lietman T, Whitcher J. Chlamydial Conjunctivitis. Ophthalmology Clinics of North America 1999;12(1):21-32.

13. Report of the Third Meeting of the W.H.O. Alliance for the Global Elimination of Trachoma. Ouarzazate, Morocco: World Health Organization, 1999.

14. Hudson AP, McEntee CM, Reacher M, et al. Inapparent ocular infection by Chlamydia trachomatis in experimental and human trachoma. Curr Eye Res 1992;11(3):279-83.

15. West S, Muñoz B, Bobo L, et al. Nonocular Chlamydia infection and risk of ocular reinfection after mass treatment in a trachoma hyperendemic area. Invest Ophthalmol Vis Sci 1993;34(11):3194-8.

16. Lode H, Borner K, Koeppe P, Schaberg T. Azithromycin--review of key chemical, pharmacokinetic and microbiological features. J Antimicrob Chemother 1996;37 Suppl C(5):1-8.

17. Tabbara K, al-Kharashi S, al-Mansouri S, et al. Ocular levels of azithromycin. Arch Ophthalmol 1998;116(12):1625-8.

18. Karma P, Pukander J, Penttilä M. Azithromycin concentrations in sinus fluid and mucosa after oral administration. Eur J Clin Microbiol Infect Dis 1991;10(10):856-9.

19. Wildfeuer A, Laufen H, Zimmermann T. Distribution of orally administered azithromycin in various blood compartments. Int J Clin Pharmacol Ther 1994;32(7):356-60.

20. Karcioglu ZA, El-Yazigi A, Jabak MH, et al. Pharmacokinetics of azithromycin in trachoma patients: serum and tear levels. Ophthalmology 1998;105(4):658-61.

21. Vaudaux BP, Cherpillod J, Dayer P. Concentrations of azithromycin in tonsilar and/or adenoid tissue from paediatric patients. J Antimicrob Chemother 1996;37 Suppl C(5):45-51.

22. Steingrímsson O, Olafsson JH, Thórarinsson H, et al. Single dose azithromycin treatment of gonorrhea and infections caused by C. trachomatis and U. urealyticum in men. Sex Transm Dis 1994;21(1):43-6.

23. Tabbara KF, Abu-el-Asrar A, al-Omar O, et al. Single-dose azithromycin in the treatment of trachoma. A randomized, controlled study. Ophthalmology 1996;103(5):842-6.

24. Bailey RL, Arullendran P, Whittle HC, Mabey DC. Randomised controlled trial of single-dose azithromycin in treatment of trachoma. Lancet 1993;342(8869):453-6.

25. Dawson CR, Schachter J, Sallam S, et al. A comparison of oral azithromycin with topical oxytetracycline/polymyxin for the treatment of trachoma in children. Clin Infect Dis 1997;24(3):363-8.

26. Stamm WE, Hicks CB, Martin DH, et al. Azithromycin for empirical treatment of the nongonococcal urethritis syndrome in men. A randomized double-blind study. JAMA 1995;274(7):545-9.

27. Erdogru T, Agaçfidan A, Onel M, et al. The treatment of non-gonococcal urethritis with single dose oral azithromycin. J Int Med Res 1995;23(5):386-93.

28. Magid D, Douglas JM, Jr., Schwartz JS. Doxycycline compared with azithromycin for treating women with genital Chlamydia trachomatis infections: an incremental cost-effectiveness analysis. Ann Intern Med 1996;124(4):389-99.

29. Thorpe EM, Jr., Stamm WE, Hook EW, 3rd, et al. Chlamydial cervicitis and urethritis: single dose treatment compared with doxycycline for seven days in community based practises. Genitourin Med 1996;72(2):93-7.

30. Hillis SD, Coles FB, Litchfield B, et al. Doxycycline and azithromycin for prevention of chlamydial persistence or recurrence one month after treatment in women. A use-effectiveness study in public health settings. Sex Transm Dis 1998;25(1):5-11.

31. Wehbeh HA, Ruggeirio RM, Shahem S, et al. Single-dose azithromycin for Chlamydia in pregnant women. J Reprod Med 1998;43(6):509-14.

32. Bianchi A, Bogard M, Cessot G, et al. Kinetics of Chlamydia trachomatis clearance in patients with azithromycin, as assessed by first void urine testing by PCR and transcription-mediated amplification. Sex Transm Dis 1998;25(7):366-7.

33. Ferriman A. Blinding trachoma almost eliminated from Morocco. Br Med J 2001;323(7326):1387.

34. Holm SO, Jha HC, Bhatta RC, et al. Comparison of two azithromycin distribution strategies for controlling trachoma in Nepal. Bull World Health Organ 2001;79(3):194-200.

35. Laming AC, Currie BJ, DiFrancesco M, et al. A targeted, single-dose azithromycin strategy for trachoma. Med J Aust 2000;172(4):163-6.

36. Schachter J, West SK, Mabey D, et al. Azithromycin in control of trachoma. Lancet 1999;354(9179):630-5.

37. Gaynor BD, Miao Y, Cevallos V, et al. Eliminating trachoma in areas with limited disease. Emerg Infect Dis 2003;9(5):596-8.

38. Equi A, Balfour-Lynn IM, Bush A, Rosenthal M. Long term azithromycin in children with cystic fibrosis: a randomised, placebo-controlled crossover trial. Lancet 2002;360(9338):978-84.

39. Marchisio P, Principi N, Sala E, et al. Comparative study of once-weekly azithromycin and once-daily amoxicillin treatments in prevention of recurrent acute otitis media in children. Antimicrob Agents Chemother 1996;40(12):2732-6.

40. Shafran SD. Prevention and treatment of disseminated Mycobacterium avium complex infection in human immunodeficiency virus-infected individuals. Int J Infect Dis 1998;3(1):39-47.

41. Dawson C. Personal communication. 2000.

42. Keshishyan H, Hanna L, Jawefz E. Emergence of rifampin-resistance in *Chlamydia trachomatis*. Nature 1973;244:173-4.

43. Stamm WE. Potential for antimicrobial resistance in Chlamydia pneumoniae. J Infect Dis 2000;181 Suppl 3(2):S456-9.

44. Dessus-Babus S, Bébéar CM, Charron A, et al. Sequencing of gyrase and topoisomerase IV quinolone-resistance-determining regions of Chlamydia trachomatis and characterization of quinolone-resistant mutants obtained In vitro. Antimicrob Agents Chemother 1998;42(10):2474-81.

45. Lefevre JC, Lepargneur JP, Guion D, Bei S. Tetracycline-resistant Chlamydia trachomatis in Toulouse, France. Pathol Biol 1997;45(5):376-8.

46. Lefèvre JC, Lépargneur JP. Comparative in vitro susceptibility of a tetracycline-resistant Chlamydia trachomatis strain isolated in Toulouse (France). Sex Transm Dis 1998;25(7):350-2.

47. Andersen A, Rogers D. Resistance to tetracycline and sulfadiazine in swine *C.trachomatis* isolates. In: Stephens Rea, ed. Ninth International Symposium on Human Chlamydial Infection. Napa, California: Ninth International Symposium on Human Chlamydial Infection, 1998.

48. Jones RB, Van der Pol B, Johnson RB. Susceptibility of Chlamydia trachomatis to trovafloxacin. J Antimicrob Chemother 1997;39 Suppl B(8):63-5.

49. Somani J, Bhullar VB, Workowski KA, et al. Multiple drug-resistant Chlamydia trachomatis associated with clinical treatment failure. J Infect Dis 2000;181(4):1421-7.

50. Goodman LS, Gilman A, Hardman JG, et al. Goodman & Gilman's the pharmacological basis of therapeutics, 9th / ed. New York: McGraw-Hill Health Professions Division, 1996; xxi, 1905.

51. McOrist S. Obligate intracellular bacteria and antibiotic resistance. Trends Microbiol 2000;8(11):483-6.

52. Adegbola RA, Mulholland EK, Bailey R, et al. Effect of azithromycin on pharyngeal microflora. Pediatr Infect Dis J 1995;14(4):335-7.

53. Leach AJ, Shelby-James TM, Mayo M, et al. A prospective study of the impact of community-based azithromycin treatment of trachoma on carriage and resistance of Streptococcus pneumoniae. Clin Infect Dis 1997;24(3):356-62.

54. Chern KC, Shrestha SK, Cevallos V, et al. Alterations in the conjunctival bacterial flora following a single dose of azithromycin in a trachoma endemic area. Br J Ophthalmol 1999;83(12):1332-5.

55. Morita JY, Kahn E, Thompson T, et al. Impact of azithromycin on oropharyngeal carriage of group A Streptococcus and nasopharyngeal carriage of macrolide-resistant Streptococcus pneumoniae. Pediatr Infect Dis J 2000;19(1):41-6.

56. Tait-Kamradt A, Davies T, Cronan M, et al. Mutations in 23S rRNA and ribosomal protein L4 account for resistance in pneumococcal strains selected in vitro by macrolide passage. Antimicrob Agents Chemother 2000;44(8):2118-25.

57. Klugman KP, Capper T, Widdowson CA, et al. Increased activity of 16-membered lactone ring macrolides against erythromycin-resistant Streptococcus pyogenes and Streptococcus pneumoniae: characterization of South African isolates. J Antimicrob Chemother 1998;42(6):729-34.

58. Shortridge VD, Doern GV, Brueggemann AB, et al. Prevalence of macrolide resistance mechanisms in Streptococcus pneumoniae isolates from a multicenter antibiotic resistance surveillance study conducted in the United States in 1994-1995. Clin Infect Dis 1999;29(5):1186-8.

59. Johnston NJ, De Azavedo JC, Kellner JD, Low DE. Prevalence and characterization of the mechanisms of macrolide, lincosamide, and streptogramin resistance in isolates of Streptococcus pneumoniae. Antimicrob Agents Chemother 1998;42(9):2425-6.

60. Gay K, Baughman W, Miller Y, et al. The emergence of Streptococcus pneumoniae resistant to macrolide antimicrobial agents: a 6-year population-based assessment. J Infect Dis 2000;182(5):1417-24.

61. Fry A, Jha H, Chaudary J, et al. Secondary effects of mass chemoprophylaxis with azithromycin to eliminate blindness due to trachoma in Nepal. Fifth Meeting of the W.H.O. Alliance for the Global Elimination of Trachoma 2000;GET/ALL5/WP5.1E.

62. Gaynor BD, Holbrook KA, Whitcher JP, et al. Community Treatment with Azithromycin for Trachoma is not Associated with Antibiotic Resistance in Streptococcus pneumoniae at One Year. Br J Ophthalmol 2003;87(2):147-8.

63. Yorke JA, Hethcote HW, Nold A. Dynamics and control of transmission of gonnorrhea. Sex Transm Dis 1978;5:51-6.

64. Jacquez J, Simon C, Koopman J. Core Groups and Ro for sub-groups in heterogenous SIS and SI models. In: Mollison D, ed. Epidemic Models: Their Structure and Relation to Data. Cambridge, UK: Cambridge University Press, 1995.

65. Courtright P, Sheppard J, Schachter J, et al. Trachoma and blindness in the Nile Delta: current patterns and projections for the future in the rural Egyptian population. Br J Ophthalmol 1989;73(7):536-40.

66. Solomon AW, Holland MJ, Burton MJ, et al. Strategies for control of trachoma: observational study with quantitative PCR. Lancet 2003;362(9379):198-204.

67. Arno JN, Katz BP, McBride R, et al. Age and clinical immunity to infections with Chlamydia trachomatis. Sex Transm Dis 1994;21(1):47-52.

68. Schachter J, Dawson C. Human Chlamydial Infections. Littleton, Massachusetts: PSG Publishing Company, Inc., 1978; 35.

69. Bailey R, Duong T, Carpenter R, et al. The duration of human ocular Chlamydia trachomatis infection is age dependent. Epidemiol Infect 1999;123(3):479-86.

70. Anderson R, May R. Infectious Diseases of Humans: Dynamics and Control. Oxford: Oxford University Press, 1991.

71. Hethcote H. Modeling heterogeneous mixing in infectious disease dynamics. In: Isham V, Medley G, eds. Modeling Heterogeneous Mixing in Infectious Disease Dynamics. Models for Infectious Human Diseases: Their Structure and Relation to Data. Cambridge: Cambridge University Press, 1996.

72. Taylor H. Towards the global elimination of trachoma. Nat Med 1999;5(5):492-3.

73. Dolin PJ, Faal H, Johnson GJ, et al. Reduction of trachoma in a sub-Saharan village in absence of a disease control programme. Lancet 1997;349(9064):1511-2.

74. Emerson PM, Cairncross S, Bailey RL, Mabey DC. Review of the evidence base for the 'F' and 'E' components of the SAFE strategy for trachoma control. Trop Med Int Health 2000;5(8):515-27.

75. West SK, Muñoz B, Lynch M, et al. Risk factors for constant, severe trachoma among preschool children in Kongwa, Tanzania. Am J Epidemiol 1996;143(1):73-8.

76. Munoz B, Aron J, Turner V, West S. Incidence estimates of late stages of trachoma among women in a hyperendemic area of central Tanzania. Trop Med Int Health 1997;2(11):1030-8.

77. Taylor HR, Rapoza PA, West S, et al. The epidemiology of infection in trachoma. Invest Ophthalmol Vis Sci 1989;30(8):1823-33.

78. West S, Muñoz B, Lynch M, et al. Impact of face-washing on trachoma in Kongwa, Tanzania. Lancet 1995;345(8943):155-8.

79. Comment. It should be noted that in a multivariate analysis the authors found that face washing was a statistically significant protective factor for severe trachomatous inflammation (TI). However the statistical significance disappears if village-clustering is taken into account or if p-values are adjusted for multiple comparisons. TI is more likely to represent infection with chlamydia, so in that sense is a more sensitive indicator than follicular trachoma (TF). However, other bacterial infections frequently cause TI, but they do not cause TF.

80. Dawson C. Flies and the elimination of blinding trachoma. Lancet 1999;353(9162):1376-7.

81. Darougar SF, T; Jones, BR; Allami, J; Houshmand, A. Isolation of Chlamydia trachomatis from eye secretion (tears). Br J Ophthalmol 1979;63(4):256-8.

82. Fifth Meeting of the W.H.O. Alliance for the Global Elimination of Trachoma. Geneva: World Health Organization, 2000.

83. Miller K, Pakpour N, Yi E, et al. Pesky trachoma suspect finally caught. British Journal of Ophthalmology 2004;In Press.

84. Emerson PM, Lindsay SW, Walraven GE, et al. Effect of fly control on trachoma and diarrhoea. Lancet 1999;353(9162):1401-3.

85. Murray DM. Design and Analysis of Group-Randomized Trials. New York: Oxford University Press, 1998.

86. Garland SM, Malatt A, Tabrizi S, et al. Chlamydia trachomatis conjunctivitis. Prevalence and association with genital tract infection. Med J Aust 1995;162(7):363-6.

87. Bailey RL, Hayes L, Pickett M, et al. Molecular epidemiology of trachoma in a Gambian village. Br J Ophthalmol 1994;78(11):813-7.

88. Bain D, Lietman T, Rasmussen S, et al. Chlamydial Genovar Distribution Following Community-Wide Antibiotic Treatment. J Infect Dis 2001;184(12):1581-8.

89. Hayes LJ, Pecharatana S, Bailey RL, et al. Extent and kinetics of genetic change in the omp1 gene of Chlamydia trachomatis in two villages with endemic trachoma. J Infect Dis 1995;172(1):268-72.

90. Dean D, Stephens RS. Identification of individual genotypes of Chlamydia trachomatis from experimentally mixed serovars and mixed infections among trachoma patients. J Clin Microbiol 1994;32(6):1506-10.

91. Dean D, Oudens E, Bolan G, et al. Major outer membrane protein variants of Chlamydia trachomatis are associated with severe upper genital tract infections and histopathology in San Francisco. J Infect Dis 1995;172(4):1013-22.

92. Dean D, Millman K. Molecular and mutation trends analyses of omp1 alleles for serovar E of Chlamydia trachomatis. Implications for the immunopathogenesis of disease. J Clin Invest 1997;99(3):475-83.

93. Bobo LD, Novak N, Muñoz B, et al. Severe disease in children with trachoma is associated with persistent Chlamydia trachomatis infection. J Infect Dis 1997;176(6):1524-30.

94. Zhang J, Lietman T, Olinger L, et al. Genetic diversity of *Chlamydia trachomatis* and the prevalence of trachoma. Pediatric Infectious Disease Journal 2004;23(3):pending.

95. Eisenberg JN, Lewis BL, Porco TC, et al. Bias due to secondary transmission in estimation of attributable risk from intervention trials. Epidemiology 2003;14(4):442-50.

96. Bernoulli D. Essai d'une nouvelle analyse de la mortalite causee par la petitie Verole, & des avantages de l'Inoculation pour la prevenir. Memoires de Mathematique et de Physique 1760:1-45.

97. Bradley L. Smallpox Inoculation: An Eighteenth Century Mathematical Controversy. Ann Arbor: University of Michigan, 1971.

98. Daley D, Gani J. Epidemic modelling: an introduction. Cambridge: Press Syndicate of the University of Cambridge, 1999.

99. Ball F. Stochastic and deterministic models for SIS epidemics among a population partitioned into households. Math Biosci 1999;156(1-2):41-67.

100. Becker NG, Dietz K. The effect of household distribution on transmission and control of highly infectious diseases. Math Biosci 1995;127(2):207-19.

101. Gupta S, Trenholme K, Anderson RM, Day KP. Antigenic diversity and the transmission dynamics of Plasmodium falciparum. Science 1994;263(5149):961-3.

102. Gupta S, Maiden MC, Feavers IM, et al. The maintenance of strain structure in populations of recombining infectious agents. Nat Med 1996;2(4):437-42.

103. Gupta S, Ferguson N, Anderson R. Chaos, persistence, and evolution of strain structure in antigenically diverse infectious agents. Science 1998;280(5365):912-5.

104. Muñoz B, West S, Aron J. Dynamics of transmission and progression of trachoma in hyperendemic areas. Annual Meeting of the Society for Mathematical Biology. Oaxtepec,Mexico, 1995.

105. Lietman T, Porco T, Dawson C, Blower S. Mass antibiotics in trachoma control: whom shall we treat and how often? In: Stephens R, Byrne G, Christiansen G, et al., eds. Chlamydial Infections: Proceedings of the Ninth International Symposium on Human Chlamydial Infection. Napa, California: International Chlamydia Symposium, 1998.

106. Baral K, Osaki S, Shreshta B, et al. Reliability of clinical diagnosis in identifying infectious trachoma in a low-prevalence area of Nepal. Bull World Health Organ 1999;77(6):461-6.

107. Thein J, Zhao P, Liu H, et al. Does clinical activity indicate ocular chlamydial infection in areas with low prevalences of trachoma? Ophthalmic Epidemiol 2002;9(4):263-9.

108. Miller K, Schmidt G, Melese M, et al. How reliable is the clinical exam in detecting ocular chlamydial infection? Ophthalmic Epidemiol 2004;in press.

109. Schachter J. Diagnosis of human chlamydial infections. In: Stephens R, ed. Ninth International Symposium on Human Chlamydial Infection. Napa, California: Ninth International Symposium on Human Chlamydial Infection, 1998.

110. Dean D, Ferrero D, McCarthy M. Comparison of performance and cost-effectiveness of direct fluorescent-antibody, ligase chain reaction, and PCR assays for verification of chlamydial enzyme immunoassay results for populations with a low to moderate prevalence of Chlamydia trachomatis infection. J Clin Microbiol 1998;36(1):94-9.

111. Dorfman. The detection of defective members of large populations. Annals of Mathematical Statistics 1943;44.

112. Keeler. Effects of Pooled Samples. Health Lab Science 1975;13(2):121-8.

113. Sacks JM, Bolin SR, Crowder SV. Prevalence estimation from pooled samples. Am J Vet Res 1989;50(2):205-6.

114. Krepel J, Patel J, Sproston A, et al. The impact on accuracy and cost of ligase chain reaction testing by pooling urine specimens for the diagnosis of Chlamydia trachomatis infections. Sex Transm Dis 1999;26(9):504-7.

115. Amos CI, Frazier ML, Wang W. DNA pooling in mutation detection with reference to sequence analysis. Am J Hum Genet 2000;66(5):1689-92.

116. Breen G, Sham P, Li T, et al. Accuracy and sensitivity of DNA pooling with microsatellite repeats using capillary electrophoresis. Mol Cell Probes 1999;13(5):359-65.

117. Kacena KA, Quinn SB, Howell MR, et al. Pooling urine samples for ligase chain reaction screening for genital Chlamydia trachomatis infection in asymptomatic women. J Clin Microbiol 1998;36(2):481-5.

118. Morré SA, Meijer CJ, Munk C, et al. Pooling of urine specimens for detection of asymptomatic Chlamydia trachomatis infections by PCR in a low-prevalence population: cost-saving strategy for epidemiological studies and screening programs. J Clin Microbiol 2000;38(4):1679-80.

119. Peeling RW, Toye B, Jessamine P, Gemmill I. Pooling of urine specimens for PCR testing: a cost saving strategy for Chlamydia trachomatis control programmes. Sexually Transmitted Infections 1998;74(1):66-70.

120. Smith R, Heldebrant C. Large scale PCR screening of pooled plasma samples for HIV-1 and HCV. Dev Biol Stand 2000;102(2):109-11.

121. Verstraeten T, Farah B, Duchateau L, Matu R. Pooling sera to reduce the cost of HIV surveillance: a feasibility study in a rural Kenyan district. Trop Med Int Health 1998;3(9):747-50.

122. Zenios SA, Wein LM. Pooled testing for HIV prevalence estimation: exploiting the dilution effect. Stat Med 1998;17(13):1447-67.

123. Kapala. Pooling cervical swabs and testing by ligase chain reaction are accurate and cost-saving strategies for diagnosis of chlamydia trachomatis. J Clin Microbiol 2000;38(7):2480-83.

124. Quinn TC, Brookmeyer R, Kline R, et al. Feasibility of pooling sera for HIV-1 viral RNA to diagnose acute primary HIV-1 infection and estimate HIV incidence. AIDS 2000;14(17):2751-7.

125. Diamont J, Moncada J, Pang F, et al. Pooling of Chlamydial Laboratory Tests to Determine Prevalence of Trachoma. Ophthalmic Epidemiol 2001;8(2-3):109-17.

126. Jones BR. The prevention of blindness from trachoma. Transactions of the Ophthalmological Societies of the United Kingdom 1975;95(1):16-33.

127. Fry AM, Jha HC, Lietman T, et al. Secondary effects of mass chemoprophylaxis with azithromycin to eliminate blindness due to trachoma in Nepal: Adverse and beneficial effects. Clin Infect Dis 2002;35(4):395-402.

128. Basilion EV, Kilima,P.M., Mecaskey JW, et al. Simplification and improvement of height - based azithromycin treatment for peadiatric trachoma TRSTMH (2005) 9: 6-12
